# Effects of imagery practice on basketball-related skills and psychological outcomes: a systematic review and Bayesian meta-analysis

**DOI:** 10.3389/fpsyg.2026.1888190

**Published:** 2026-07-31

**Authors:** Zhiming Shan, Weiqian Yang, Pu Wang

**Affiliations:** 1 Nanjing Sport Institute, Nanjing, Jiangsu, China; 2 Jinling Institute of Technology, Nanjing, Jiangsu, China; 3 Southern University of Science and Technology, Shenzhen, Guangdong, China

**Keywords:** basketball, Bayesian multilevel meta-analysis, free throw performance, imagery practice, shooting performance

## Abstract

**Background:**

Imagery practice is widely used in basketball training. Its effects on basketball-related skill and psychological outcomes remain uncertain because most trials are small and assess heterogeneous outcomes.

**Objective:**

This meta-analysis examined the effects of imagery practice on basketball-related skill and psychological outcomes.

**Methods:**

Randomized controlled trials were included when they compared imagery practice with usual training or a control condition without imagery. Hedges’ g was calculated from change scores. Positive values indicated better outcomes after imagery practice. Bayesian multilevel models were used to account for dependent effect sizes arising from multiple outcomes, intervention arms, or testing conditions within the same study, thereby reducing the risk of inflated precision when clustered sport-imagery effects are treated as independent. Outcome subgroup analyses, population subgroup analyses, duration models, publication bias tests, model diagnostics, and GRADE assessment were conducted.

**Results:**

The analysis included 39 effect sizes from 14 study units. Across basketball-related skill and psychological outcomes, imagery practice showed a positive overall effect, Hedges’ g, 1.08, 95% HDI, 0.65 to 1.52, BF10, 317.67. The clearest outcome evidence was found for free throw performance, Hedges’ g, 0.89, 95% HDI, 0.38 to 1.40, BF10, 13.85. Positive effects were also estimated for motor coordination control and self-efficacy, but these outcomes were based on fewer studies. Evidence was imprecise for basketball shooting accuracy, free throw performance under time pressure, and three point shooting because the 95% HDI crossed zero. Egger regression suggested small study effects. Model diagnostics were acceptable, with Rhat values close to 1.00.

**Conclusion:**

Imagery practice may improve basketball-related outcomes, with the most defensible evidence observed for free throw performance. Evidence for motor coordination/control and self-efficacy should be interpreted as secondary and indirect support rather than direct evidence of basketball skill improvement. Current evidence remains limited by small samples, clustered effects, and possible small-study effects. Future trials should use larger samples, clearer allocation methods, and standardized basketball skill outcomes.

**Systematic review registration:**

https://www.crd.york.ac.uk/PROSPERO/view/CRD420261389048.

## Introduction

Basketball performance depends on more than physical capacity (Petway et al., 2020). Players must coordinate movement, attention, timing, and emotional control during repeated skill execution (Petway et al., 2020; Ashford et al., 2021). Shooting is especially sensitive to these demands. A free throw has a stable task structure, yet performance may still decline when attention, pressure, or fatigue disrupts movement control (Wilson et al., 2009). Other basketball skills involve greater environmental variability. Passing, dribbling, and shooting in game-like situations require rapid perception, accurate decision-making, and precise motor execution (Ashford et al., 2021). These characteristics make basketball a useful context for examining psychological training methods that aim to improve sport-specific performance (Simonsmeier et al., 2021).

Imagery practice is used in this review as the preferred umbrella term for structured cognitive rehearsal of movements or performance situations without overt physical execution (Toth et al., 2020). Its theoretical basis is linked to the overlap between imagined action and motor preparation (Jeannerod, 2001). When an athlete imagines a basketball shot, the athlete may mentally rehearse the movement sequence, body position, rhythm, visual target, and expected outcome. This process may help refine motor representations (Di Rienzo et al., 2016). It may also support attentional control, confidence, and preparation for performance demands (Guillot and Collet, 2008). These mechanisms are relevant to basketball because many skills depend on stable routines, precise timing, and the ability to reproduce coordinated actions under variable conditions (Ashford et al., 2021; Goldschmied et al., 2022). These processes may also support movement representation, neuromuscular preparation, pre-performance routines, and quiet-eye or gaze-control preparation, although the included trials rarely measured these mechanisms directly.

Previous sport imagery and mental-practice meta-analyses have generally reported beneficial effects on sport performance and have examined broad moderators such as task characteristics, control condition, imagery format, and practice dosage (Simonsmeier et al., 2021; Toth et al., 2020). More recent Bayesian and multilevel syntheses also suggest that imagery practice may improve athletic performance and selected psychological outcomes, including self-efficacy and confidence, although these reviews pooled across sports and outcome families rather than isolating basketball-specific skill evidence (Liu et al., 2025; Zhang et al., 2025). Recent sport-specific imagery trials also illustrate the broader intervention landscape beyond basketball. For example, PETTLEP imagery has been used to improve football passing skill acquisition in novice athletes, and functional imagery training has been examined for motor performance and well-being outcomes in elite handball players after COVID-19. These studies support the wider relevance of structured imagery-based interventions in sport, while also highlighting the need to interpret effects within the specific task and sport context (Norouzi et al., 2025; Norouzi et al., 2019). These syntheses provide the broader empirical basis for imagery practice, but they aggregate evidence across multiple sports, populations, and outcome families. Therefore, they do not directly show whether the evidence is equally defensible within a single sport such as basketball, where standardized closed skills, such as free throw shooting, coexist with more open, distance-based, or pressure-sensitive shooting outcomes. In basketball, many studies have focused on free throw shooting because it is standardized, repeatable, and easy to quantify (Goldschmied et al., 2022). Other studies have examined shooting accuracy, basketball-specific skill outcomes, and performance-relevant psychological outcomes (Fazel et al., 2018). This evidence suggests that imagery practice may be useful for basketball players (Fazel et al., 2018). Recent sport-specific imagery trials also illustrate the broader intervention landscape beyond basketball. For example, PETTLEP imagery has been used to improve football passing skill acquisition in novice athletes, and functional imagery training has been examined for motor performance and well-being outcomes in elite handball players after COVID-19. These studies support the wider relevance of structured imagery-based interventions in sport, while also highlighting the need to interpret effects within the specific task and sport context (Liu et al., 2025; Zhang et al., 2025). These syntheses provide the broader empirical basis for imagery practice, but they aggregate evidence across multiple sports, populations, and outcome families. Therefore, they do not directly show whether the evidence is equally defensible within a single sport such as basketball, where standardized closed skills, such as free throw shooting, coexist with more open, distance-based, or pressure-sensitive shooting outcomes. In basketball, many studies have focused on free throw shooting because it is standardized, repeatable, and easy to quantify (Goldschmied et al., 2022). Other studies have examined shooting accuracy, basketball-specific skill outcomes, and performance-relevant psychological outcomes (Norouzi et al., 2025). This evidence suggests that imagery practice may be useful for basketball players (Norouzi et al., 2025). It also indicates that effects may vary across outcomes (Simonsmeier et al., 2021; Toth et al., 2020). More standardized skills, such as free throw shooting, may produce more consistent estimates than open or complex basketball skills that involve greater contextual variation. However, these prior syntheses do not resolve the methodological problem created when several clustered basketball outcomes are extracted from the same randomized trial.

The current evidence remains difficult to interpret. Many studies have small samples. Some studies report multiple outcomes from the same participants. Intervention duration, imagery format, participant level, and control condition also differ across trials (Simonsmeier et al., 2021; Toth et al., 2020). These features may produce unstable estimates and create statistical dependence among effect sizes (Van den Noortgate et al., 2013). A narrative synthesis has limited ability to quantitatively distinguish an overall effect from outcome-specific patterns (Campbell et al., 2020). Conventional frequentist meta-analyses are often forced to average multiple outcomes, select a single outcome, or treat related effects as independent; each option can either discard basketball-specific information or understate uncertainty (Van den Noortgate et al., 2013). These limitations support the need for a focused Bayesian multilevel synthesis that can retain clustered effects while modelling their dependence (Van den Noortgate et al., 2013).

These features may produce unstable estimates and create statistical dependence among effect sizes (Norouzi et al., 2019). A narrative synthesis has limited ability to quantitatively distinguish an overall effect from outcome-specific patterns (Fazel et al., 2018). Conventional frequentist meta-analyses are often forced to average multiple outcomes, select a single outcome, or treat related effects as independent; each option can either discard basketball-specific information or understate uncertainty (Norouzi et al., 2019). These limitations support the need for a focused Bayesian multilevel synthesis that can retain clustered effects while modelling their dependence (Norouzi et al., 2019).

Building on prior sport imagery meta-analyses, this study examined the effects of imagery practice on basketball-related skill and psychological outcomes using a Bayesian multilevel meta-analysis. The incremental contribution was threefold. First, the review focused on basketball rather than pooling across multiple sports, allowing the evidence to be interpreted within a single sport-specific task context. Second, the analyses separated direct basketball skill outcomes from related motor-control and psychological outcomes, with free throw performance treated as the key basketball-specific outcome because it was the most frequently reported standardized skill outcome. Third, the Bayesian multilevel model retained multiple eligible effects while accounting for their clustering within research sources and study units, which is important because several basketball imagery studies reported multiple outcomes, intervention arms, or testing conditions. Secondary analyses examined whether effects differed across outcome categories, populations, and intervention duration. Publication bias, model diagnostics, and certainty of evidence were assessed to support a cautious interpretation of the findings. This design also foregrounds the applied contribution of separating self-paced free throws from open-skill, distance-based, or pressure-sensitive shooting conditions.

## Method

This research was registered with PROSPERO (CRD420261389048) and complied with the PRISMA reporting guideline (Page et al., 2020a). The Supplementary materials are cited consistently as follows: Supplementary File S1 provides the completed PRISMA 2020 checklist; Supplementary File S2 provides the complete database-specific search strategies; Supplementary File S3 provides the extracted data and effect-size dataset; Supplementary File S4 provides the RoB 2.0 domain-level judgments, numerical risk-of-bias summaries, and the self-efficacy risk-of-bias summary; Supplementary File S5 provides the Egger-type regression and publication-bias diagnostics; and Supplementary File S6 provides the GRADE summary-of-findings tables. Supplementary File S7 provides MCMC numerical diagnostics, trace plots, posterior density plots, and bulk/tail ESS diagnostics. A Bayesian meta-analysis was conducted following a systematic review utilizing Covidence, Python version 3, GRADEprofiler, R version 4.5.1, and GetData Graph Digitizer. The PRISMA flow diagram was generated using the PRISMA2020 R package (Haddaway et al., 2022).

### Eligibility criteria

This systematic review and meta-analysis was registered in PROSPERO, CRD420261389048, and was reported in accordance with PRISMA 2020 guidance (Page et al., 2020b). Eligible studies were randomized controlled trials that examined the effects of imagery practice on basketball-related outcomes. Participants were basketball players or basketball participants, including elite players, trained players, recreational players, novice players, and student samples.

Eligible interventions included imagery practice and interventions described by source authors as motor imagery, mental imagery training, visualization, PETTLEP imagery, psychological procedure training with an imagery component, and related structured imagery procedures. These intervention labels were considered eligible when they involved systematic cognitive rehearsal of a movement, skill, or performance situation without overt physical execution, consistent with established definitions of mental practice and motor imagery practice. This research was registered with PROSPERO (CRD420261389048) and complied with the PRISMA reporting guideline (Van den Noortgate et al., 2013). The Supplementary materials are cited consistently as follows: Supplementary File S1 provides the completed PRISMA 2020 checklist; Supplementary File S2 provides the complete database-specific search strategies; Supplementary File S3 provides the extracted data and effect-size dataset; Supplementary File S4 provides the RoB 2.0 domain-level judgments, numerical risk-of-bias summaries, and the self-efficacy risk-of-bias summary; Supplementary File S5 provides the Egger-type regression and publication-bias diagnostics; and Supplementary File S6 provides the GRADE summary-of-findings tables. A Bayesian meta-analysis was conducted following a systematic review utilizing Covidence, Python version 3, GRADEprofiler, R version 4.5.1, and GetData Graph Digitizer.

### Eligibility criteria

This systematic review and meta-analysis was registered in PROSPERO, CRD420261389048, and was reported in accordance with PRISMA 2020 guidance (Campbell et al., 2020). Eligible studies were randomized controlled trials that examined the effects of imagery practice on basketball-related outcomes. Participants were basketball players or basketball participants, including elite players, trained players, recreational players, novice players, and student samples.

Eligible interventions included imagery practice and interventions described by source authors as motor imagery, mental imagery training, visualization, PETTLEP imagery, psychological procedure training with an imagery component, and related structured imagery procedures. These intervention labels were considered eligible when they involved systematic cognitive rehearsal of a movement, skill, or performance situation without overt physical execution, consistent with established definitions of mental practice and motor imagery practice (Toth et al., 2020). PETTLEP imagery was included because it is a structured sport imagery approach based on functional equivalence between imagined and actual performance conditions (Holmes and Collins, 2001). Imagery practice could be delivered alone or combined with regular basketball training, physical practice, action observation, relaxation, or other psychological training components, provided that imagery was a planned and explicit component and the comparator did not include imagery. When additional non-imagery components were not matched between arms, the effect was retained but interpreted as an imagery-containing intervention effect rather than an isolated imagery effect. This decision was consistent with previous sport imagery reviews showing that imagery interventions are commonly delivered either alone or in combination with physical practice or other training components. PETTLEP imagery was included because it is a structured sport imagery approach based on functional equivalence between imagined and actual performance conditions (Page et al., 2020a). Imagery practice could be delivered alone or combined with regular basketball training, physical practice, action observation, relaxation, or other psychological training components, provided that imagery was a planned and explicit component and the comparator did not include imagery. When additional non-imagery components were not matched between arms, the effect was retained but interpreted as an imagery-containing intervention effect rather than an isolated imagery effect. This decision was consistent with previous sport imagery reviews showing that imagery interventions are commonly delivered either alone or in combination with physical practice or other training components (Simonsmeier et al., 2021).

Eligible comparators included no imagery practice, usual basketball training, no-practice control, physical practice alone, skill training alone, or other non-imagery control conditions. Studies were included when they reported quantitative outcome data that allowed the calculation of a standardized effect size. Standardized mean differences were used because the included studies assessed related performance constructs using different measurement scales or task formats. Studies were excluded when the intervention did not contain an imagery component, when the sample was unrelated to basketball performance, when random allocation was not used, when no eligible comparator was available, or when the data were insufficient for effect size calculation.

Eligible outcomes were classified according to their proximity to basketball skill execution. Primary/direct basketball skill outcomes required performance of a basketball shooting task and included free throw performance, basketball shooting accuracy, three-point shooting, and free throw performance under time pressure. Related motor-control outcomes included motor coordination or control tasks that were directly linked to basketball skill execution or movement control. Psychological outcomes included self-efficacy, which was treated as a psychological construct relevant to performance rather than as direct performance evidence (Bandura, 1977). Psychological outcomes included self-efficacy, which was treated as a psychological construct relevant to performance rather than as direct performance evidence (Page et al., 2020b). Motor-control and self-efficacy outcomes were retained as secondary basketball-related outcomes because they measured possible mechanisms or performance-related constructs rather than direct basketball skill execution. All eligible outcomes contributed to the overall model; the distinction was used to guide interpretation and GRADE prioritization.

### Information sources

A systematic search was conducted in EBSCO, PubMed, Scopus, and Web of Science from database inception to April 10, 2026, with no publication-date restriction. Search terms combined basketball-related terms, imagery or psychological-intervention terms, and performance or skill-outcome terms. Reference lists of included studies and relevant reviews were also checked to identify additional eligible records. Separate grey-literature searches of theses or dissertations, conference proceedings, and trial registries were not conducted. The complete database-specific search strings, Boolean logic, and search-date information are provided in Supplementary File S2.

### Study selection and data collection

All retrieved records were imported into reference management software, and duplicates were removed. Two reviewers independently screened titles and abstracts. Full texts of potentially eligible studies were then independently assessed by two reviewers according to the eligibility criteria. Disagreements at both the title and abstract screening stage and the full-text screening stage were first checked against the predefined eligibility criteria and then resolved through discussion between the two reviewers. If consensus could not be reached, a third reviewer adjudicated the final decision.

Data extraction was also conducted independently by two reviewers using a standardized extraction form. Extracted information included study identification, randomized trial design, sample characteristics, population level, intervention characteristics, comparator characteristics, intervention duration, outcome name, outcome subgroup, sample size, change mention, and information required for effect size calculation. When one study reported multiple eligible intervention arms, comparator arms, or outcomes, each eligible comparison was retained as a separate effect size. Dependence among multiple effect sizes from the same study was addressed in the multilevel meta-analysis.

### Data items

For each eligible comparison, we extracted or calculated baseline-to-post-intervention change scores for the imagery practice group and the comparator group. The primary numerical items were experimental group change mean, experimental group change standard deviation, control group change mean, control group change standard deviation, experimental group sample size, control group sample size, intervention duration in weeks, population level, outcome name, and outcome subgroup.

The main effect size was Hedges’ g, calculated from the difference in change scores between the imagery practice group and the comparator group. The numerator was the between-group change contrast, *Δ* = (Mpost, imagery − Mbaseline, imagery) − (Mpost, control − Mbaseline, control), standardized by the pooled change-score SD and then multiplied by Hedges’ small-sample correction. Thus, baseline mean differences were removed from the numerator at the aggregate level before standardization. No further ANCOVA-type baseline adjustment was applied because individual participant data and consistent baseline–post covariance information were unavailable; residual baseline-imbalance concerns were considered in RoB 2.0 and GRADE. This standardized effect-size metric was selected because the included studies assessed basketball-related outcomes using different task formats and measurement scales. Standardized mean differences are recommended when studies assess related continuous outcomes using different scales or units. Sampling variance and standard error were calculated for each effect size before synthesis, consistent with standard meta-analytic practice for continuous outcomes. Outcomes were coded so that positive values favored imagery practice. When lower values indicated better performance, the direction was reversed before synthesis.

Outcome subgroups were harmonized before analysis. The final categories were free throw performance, basketball shooting accuracy, three-point shooting, free throw performance under time pressure, motor coordination/control, and self-efficacy. In reporting, free throw performance, basketball shooting accuracy, three-point shooting, and free throw performance under time pressure were treated as direct basketball skill outcomes. Motor coordination/control and self-efficacy were treated as secondary basketball-related outcomes. Population levels were categorized as elite, trained, recreational, novice, or student according to the original study descriptions. The extracted data and effect-size dataset used for the meta-analysis are provided in Supplementary File S3.

### Risk of bias assessment

Risk of bias was assessed independently by two reviewers using the Cochrane revised risk-of-bias tool for randomized trials, RoB 2.0. Because RoB 2.0 is result specific, judgments were made at the outcome level rather than only at the study level. We used the effect of assignment to the intervention as the effect of interest. Six prespecified reporting domains were extracted for each eligible result: randomization process, allocation concealment, intervention delivery, outcome blinding and outcome measurement, attrition, and selective reporting. These domains were mapped to the RoB 2.0 framework (Sterne et al., 2019). These domains were mapped to the RoB 2.0 framework (Holmes and Collins, 2001). Allocation concealment was reported separately because it was a recurring source of uncertainty in the included sport trials. Intervention delivery corresponded to deviations from intended interventions. Attrition corresponded to missing outcome data. Selective reporting corresponded to selection of the reported result.

Because participant and provider blinding is generally not feasible in imagery practice studies, lack of blinding was not automatically judged as high risk of bias for objective basketball outcomes. For objective outcomes such as free throw performance, shooting accuracy, three point shooting, free throw performance under time pressure, and motor coordination or control, greater concern was assigned only when lack of blinding could plausibly change cointerventions, training exposure, encouragement, adherence, outcome measurement, or analysis. For subjective self-efficacy, lack of participant blinding was weighted more seriously because expectancy and demand characteristics could directly influence self-reported responses. Outcome blinding was also judged more strictly for self-efficacy than for objective shooting outcomes because the participant was the outcome assessor in self-report measurement.

Domain-level judgments were rated as low risk of bias, some concerns, or high risk of bias. Overall judgments were derived separately for objective basketball-related outcomes and for self-efficacy. Written justifications were recorded for each domain. The main-text risk-of-bias figure presents the outcome-specific assessment for objective basketball-related outcomes. The self-efficacy risk-of-bias figure and detailed judgments are provided in Supplementary File S4.

### Certainty in evidence

The certainty of evidence was assessed using the GRADE approach. Certainty was evaluated for all predefined key outcome subgroups across five domains: risk of bias, inconsistency, indirectness, imprecision, and publication bias. The key outcomes were free throw performance, basketball shooting accuracy, free throw performance under time pressure, three-point shooting, motor coordination/control, and self-efficacy. Randomized trials started as high-certainty evidence, consistent with GRADE guidance for intervention evidence. Certainty was downgraded when there were serious concerns about study limitations, unexplained heterogeneity, indirect population, intervention, comparator, or outcome evidence, imprecise estimates, sparse evidence, or potential publication bias.

Free throw performance was treated as the critical direct basketball-specific outcome because it was the most frequently reported standardized basketball skill. Basketball shooting accuracy, free throw performance under time pressure, and three-point shooting were treated as additional direct basketball skill outcomes. Motor coordination/control and self-efficacy were treated as secondary basketball-related outcomes because they measured related motor-control or psychological constructs rather than direct basketball skill execution. Complete summary-of-findings tables were prepared for all key outcomes. Each table reports the number of study units and effect estimates, participant observations contributing to the contrasts, posterior effect estimate, GRADE certainty rating, judgments for all five GRADE domains, reasons for downgrading, and interpretation. The full GRADE summary-of-findings tables are provided in Supplementary File S6.

### Summary measures and synthesis

The primary summary measure was Hedges’ g, which was used to correct standardized mean differences for small-sample bias (Hedges, 1981). A positive value indicated that imagery practice produced greater improvement than the comparator. The main synthesis used a Bayesian multilevel random-effects meta-analysis implemented in R 4.5.1 with the brms package (Bürkner, 2017). This modelling strategy was selected because the eligible evidence base contained multiple effect sizes from the same research sources or study units, including effects derived from different outcomes, intervention arms, or testing conditions. A single-level random-effects model would require averaging or discarding eligible effects, or treating correlated effects as independent; each option could reduce outcome-specific information or overstate precision. The Bayesian multilevel framework allowed all eligible effects to be retained while modelling clustering and uncertainty explicitly. The observed effect size and its standard error were entered into the model using known sampling error, with an additional residual heterogeneity term estimated through sigma.

The main model estimated the overall effect of imagery practice. Because the overall model included both direct basketball skill outcomes and secondary basketball-related outcomes, it was interpreted as the overall effect on basketball-related skill and psychological outcomes, rather than as a pure estimate of direct basketball performance. The main model was specified as g_i | se(SE_i, sigma = TRUE) ~ 1 + (1 | author/study), where g_i represents Hedges’ g and SE_i represents its standard error. In brms notation, (1 | author/study) expands to (1 | author) + (1 | author:study), representing random intercepts for author/source and for study units nested within author/source. This study-within-author structure was used to account for dependence among multiple effect sizes derived from the same research source or study unit, following the rationale of multilevel meta-analysis for handling dependent effect sizes (Cheung, 2014).

All Bayesian models used weakly informative priors on the standardized effect-size scale. For the overall intercept, a Normal(0, 1.5) prior was used. Because all effects were expressed as Hedges’ g, this prior placed approximately 95% of its prior mass between −2.94 and 2.94, allowing small, moderate, and large intervention effects while mildly regularizing implausibly extreme standardized effects. Group-level standard deviations and the residual heterogeneity parameter sigma used Exponential(1) priors. These priors assigned greater prior density to smaller heterogeneity values while retaining positive support for larger heterogeneity values. They were therefore selected to stabilize estimation in a relatively small evidence base with dependent effect sizes, without forcing the posterior toward a particular intervention effect, consistent with common recommendations for Bayesian hierarchical models (Gelman, 2006).

The primary summary measure was Hedges’ g, which was used to correct standardized mean differences for small-sample bias (Bandura, 1977). A positive value indicated that imagery practice produced greater improvement than the comparator. The main synthesis used a Bayesian multilevel random-effects meta-analysis implemented in R 4.5.1 with the brms package (Sterne et al., 2019). This modelling strategy was selected because the eligible evidence base contained multiple effect sizes from the same research sources or study units, including effects derived from different outcomes, intervention arms, or testing conditions. A single-level random-effects model would require averaging or discarding eligible effects, or treating correlated effects as independent; each option could reduce outcome-specific information or overstate precision. The Bayesian multilevel framework allowed all eligible effects to be retained while modelling clustering and uncertainty explicitly. The observed effect size and its standard error were entered into the model using known sampling error, with an additional residual heterogeneity term estimated through sigma.

The main model estimated the overall effect of imagery practice. Because the overall model included both direct basketball skill outcomes and secondary basketball-related outcomes, it was interpreted as the overall effect on basketball-related skill and psychological outcomes, rather than as a pure estimate of direct basketball performance. The main model was specified as g_i | se(SE_i, sigma = TRUE) ~ 1 + (1 | author/study), where g_i represents Hedges’ g and SE_i represents its standard error. In brms notation, (1 | author/study) expands to (1 | author) + (1 | author:study), representing random intercepts for author/source and for study units nested within author/source. This study-within-author structure was used to account for dependence among multiple effect sizes derived from the same research source or study unit, following the rationale of multilevel meta-analysis for handling dependent effect sizes (Hedges, 1981).

All Bayesian models used weakly informative priors on the standardized effect-size scale. For the overall intercept, a Normal(0, 1.5) prior was used. Because all effects were expressed as Hedges’ g, this prior placed approximately 95% of its prior mass between −2.94 and 2.94, allowing small, moderate, and large intervention effects while mildly regularizing implausibly extreme standardized effects. Group-level standard deviations and the residual heterogeneity parameter sigma used Exponential(1) priors. These priors assigned greater prior density to smaller heterogeneity values while retaining positive support for larger heterogeneity values. They were therefore selected to stabilize estimation in a relatively small evidence base with dependent effect sizes, without forcing the posterior toward a particular intervention effect, consistent with common recommendations for Bayesian hierarchical models (Bürkner, 2017).

Outcome subgroup and population subgroup models were fitted without an intercept, so that each subgroup coefficient directly represented the estimated effect for that subgroup. The outcome subgroup model was specified as g_i | se(SE_i, sigma = TRUE) ~ 0 + outcome_subgroup + (1 | author/study). The population subgroup model was specified as g_i | se(SE_i, sigma = TRUE) ~ 0 + population_level + (1 | author/study). These models used Normal(0, 1.5) priors for subgroup coefficients, Exponential(1) priors for group-level standard deviations, and an Exponential(1) prior for sigma.

All Bayesian models were fitted with four chains, 10,000 iterations per chain, 5,000 warmup iterations, and seed 20260504. Prior sampling was enabled. Hamiltonian Monte Carlo settings used an adapt_delta value of 0.999 and a max_treedepth value of 15. Model convergence and sampling efficiency were evaluated using both numerical and graphical MCMC diagnostics. Numerical diagnostics included Rhat, bulk effective sample size, and tail effective sample size. Graphical diagnostics included trace plots and chain-wise posterior density plots for the monitored model parameters. For each fitted Bayesian model, we inspected chain mixing, stationarity, and overlap across chains, and summarized Rhat, bulk ESS, and tail ESS for the population-level coefficients, group-level standard deviations, smooth-term standard deviations where applicable, and the residual heterogeneity parameter. Full numerical diagnostics and diagnostic plots are provided in Supplementary File S7.

Posterior estimates were summarized using the posterior mean and 95% highest density interval (95% HDI). Bayes factors were calculated from prior and posterior distributions using bayestestR and reported as BF10 or log_BF (Makowski et al., 2019; Cheung, 2014). BF10 was interpreted as evidence for the alternative hypothesis relative to a point-null hypothesis. The following thresholds were defined *a priori* for interpretation: BF10 values between 1/3 and 3 were considered weak or inconclusive evidence; values from 3 to 10 were considered moderate evidence; values from 10 to 30 were considered strong evidence; values from 30 to 100 were considered very strong evidence; and values greater than 100 were considered extreme evidence for the alternative hypothesis. BF10 values below 1/3 were considered evidence favoring the point-null hypothesis. Bayes factors were treated as supplementary evidence and were interpreted alongside posterior effect estimates and 95% HDIs.

Small-study effects and publication bias were explored using contour-enhanced funnel plots and Egger-type regression (Egger et al., 1997). These analyses were interpreted cautiously because several studies contributed multiple effect sizes and conventional funnel plot and Egger-type methods assume independent effect estimates. Under dependent effect-size conditions, the effective amount of independent information may be smaller than the nominal number of effects, which can reduce the statistical power of conventional Egger regression to detect small-study effects and can also complicate interpretation of funnel asymmetry (Rodgers and Pustejovsky, 2021).

Small-study effects and publication bias were explored using contour-enhanced funnel plots and Egger-type regression (Gelman, 2006). These analyses were interpreted cautiously because several studies contributed multiple effect sizes and conventional funnel plot and Egger-type methods assume independent effect estimates. Under dependent effect-size conditions, the effective amount of independent information may be smaller than the nominal number of effects, which can reduce the statistical power of conventional Egger regression to detect small-study effects and can also complicate interpretation of funnel asymmetry (Makowski et al., 2019).

### Moderation analysis

Prespecified moderation analyses examined whether the effect of imagery practice differed by outcome subgroup, population level, and intervention duration. Outcome subgroup models compared free throw performance, basketball shooting accuracy, three-point shooting, free throw performance under time pressure, motor coordination or control, and self-efficacy. Population subgroup models compared elite, trained, recreational, novice, and student samples.

Intervention duration was examined as a continuous moderator using Bayesian generalized additive models. Duration was coded in weeks. The duration model was specified as g_i | se(SE_i, sigma = TRUE) ~ 1 + s(duration_weeks, k = 5) + (1 | author/study). The smooth term was fitted as a penalized smooth function. The basis dimension was constrained to k = 5 because the number of unique duration values was limited and the evidence base was small. This constraint allowed only a low-complexity non-linear association and reduced the risk of overfitting. The smooth term used the default identifiability constraints implemented by the modelling software, and the smoothing penalty was estimated within the Bayesian model. The same study-within-author random-intercept structure, (1 | author/study), was used in the duration models to account for dependence among effect sizes from the same research source or study unit.

The duration models used a Normal(0, 1.5) prior for the intercept, an Exponential(1) prior for the smooth-term standard deviation, Exponential(1) priors for group-level standard deviations, and an Exponential(1) prior for sigma, consistent with the prior structure used in the main Bayesian models (Gelman, 2006; Bürkner, 2017). Duration models were fitted for the overall dataset and for outcome subgroups when there were sufficient effect sizes and sufficient variation in duration. When the number of effects or the number of unique duration values was insufficient, the subgroup duration model was not fitted. Predictions were not extrapolated beyond the observed intervention-duration range. All moderation analyses, including the population subgroup analyses, were prespecified exploratory analyses intended to describe possible sources of heterogeneity. Because several population categories were represented by few study units or effect estimates, subgroup estimates were interpreted as preliminary and hypothesis-generating and were not used as definitive evidence of differential intervention effects.

## Results

The results include six parts, namely study selection, the characteristics of included studies, quality assessment, meta-analysis, moderation analysis, quality grade and publication bias.

### Study selection

A total of 5,768 records were identified from four databases: EBSCO (*n* = 1,702), PubMed (*n* = 716), Scopus (*n* = 2,050), and Web of Science (*n* = 1,300). After removing 1,775 duplicate records, 3,993 records were screened by title and abstract in ASReview. Of these, 3,820 records were excluded. The remaining 173 reports were sought for retrieval, and all were obtained.

**Figure 1:**
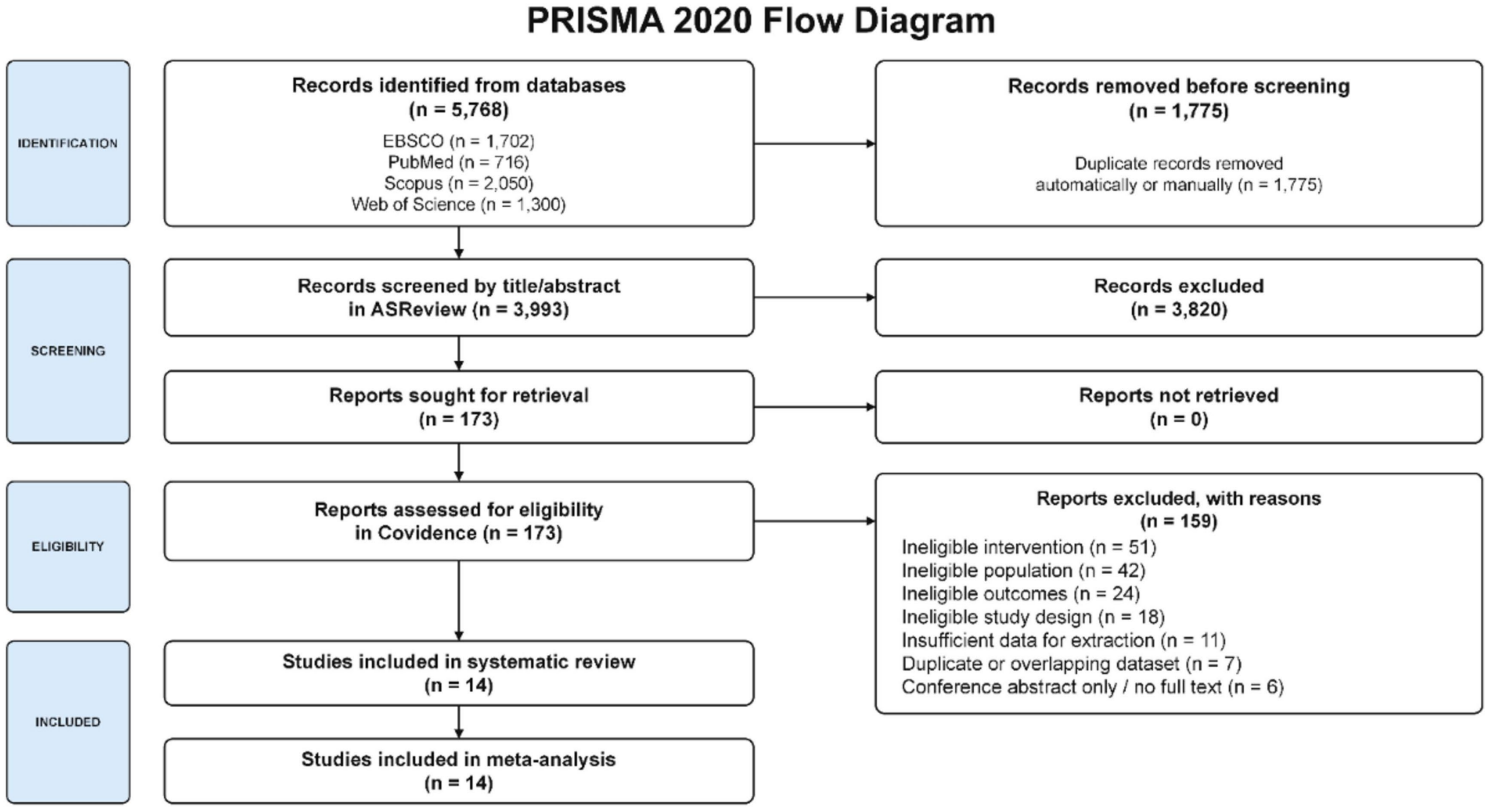
PRISMA flow chart for the identification of the included studies.

A total of 173 full-text reports were assessed for eligibility in Covidence. Overall, 159 reports were excluded. The main reasons were ineligible intervention (*n* = 51), ineligible population (*n* = 42), ineligible outcomes (*n* = 24), ineligible study design (*n* = 18), insufficient data for extraction (*n* = 11), duplicate or overlapping dataset (*n* = 7), and conference abstract only or no full text (*n* = 6). Finally, 14 studies were included in the systematic review and meta-analysis. The study selection process is shown in Figure 1.

### Characteristics of included studies

The meta-analysis included 14 studies and 39 effect estimates. Several studies contributed more than one effect estimate because they reported multiple outcomes, intervention arms, or testing conditions. They included samples covered elite, trained, recreational, novice, and student basketball populations. In the extracted effect-size dataset, novice participants contributed the largest number of effects, followed by elite participants, trained participants, recreational participants, and student participants.

The most common direct basketball skill outcome was free throw performance. Other direct basketball skill outcomes included basketball shooting accuracy, three-point shooting, and free throw performance under time pressure. Motor coordination/control and self-efficacy were included as secondary basketball-related outcomes. Intervention duration ranged from acute or immediate testing to 8 weeks, with a median duration of 4 weeks. Where age was reported, mean age ranged from 11.60 to 25.36 years.

### Quality assessment

The RoB 2.0 assessment showed that the main methodological concerns were related to randomization reporting, allocation concealment, intervention delivery, and selective reporting. For objective basketball-related outcomes, lack of participant and provider blinding was considered unavoidable in imagery practice interventions and was not automatically judged as high risk of bias. Outcome measurement was generally less concerning for objective outcomes when shooting performance or motor-control performance was assessed using standardized tasks, numerical scoring, or clearly defined performance criteria. However, studies were rated as having some concerns when randomization, allocation concealment, adherence, cointerventions, or selective reporting were insufficiently described.

**Figure 2:**
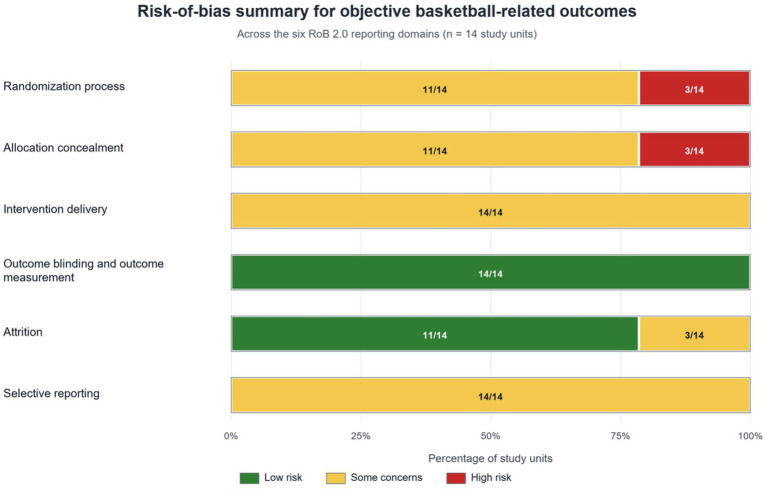
Risk-of-bias summary for objective basketball-related outcomes across the six RoB 2.0 reporting domains.

The risk-of-bias profile was less favorable for self-efficacy outcomes. Because self-efficacy was measured using self-report, participant awareness of the intervention could directly influence outcome responses through expectancy or demand effects. Therefore, intervention delivery and outcome blinding were weighted more seriously for self-efficacy than for objective basketball skill outcomes. The RoB 2.0 summary for objective basketball-related outcomes is shown in Figure 2. The accompanying numerical summary across the six RoB 2.0 reporting domains was as follows: randomization process, 0/14 low risk, 11/14 some concerns, and 3/14 high risk; allocation concealment, 0/14 low risk, 11/14 some concerns, and 3/14 high risk; intervention delivery, 0/14 low risk, 14/14 some concerns, and 0/14 high risk; outcome blinding and outcome measurement, 14/14 low risk, 0/14 some concerns, and 0/14 high risk; attrition, 11/14 low risk, 3/14 some concerns, and 0/14 high risk; and selective reporting, 0/14 low risk, 14/14 some concerns, and 0/14 high risk. Other sources of bias, if retained, were treated only as a supplementary methodological consideration in Supplementary File S4 and were not counted as one of the six RoB 2.0 reporting domains. The self-efficacy risk-of-bias summary figure and detailed domain-level judgments are provided in Supplementary File S4.

### The results of meta-analysis

Bayesian multilevel meta-analysis was performed using Hedges’ g as the effect measure. Across the fitted Bayesian models, MCMC convergence and sampling efficiency were acceptable. The maximum Rhat was 1.002, the minimum bulk effective sample size was 3,904.71, and the minimum tail effective sample size was 6810.04. Visual inspection showed well-mixed chains without sustained trends in the trace plots, and chain-wise posterior density plots showed similar posterior distributions across chains. All reported bulk and tail ESS values exceeded the reference value of 400. Full parameter-level Rhat, bulk ESS, and tail ESS values are reported in Supplementary Table S7.1, and the trace plots, chain-wise posterior density plots, and bulk/tail ESS plots are shown in Supplementary Figures S7.1–S7.12.

### Overall model

**Figure 3:**
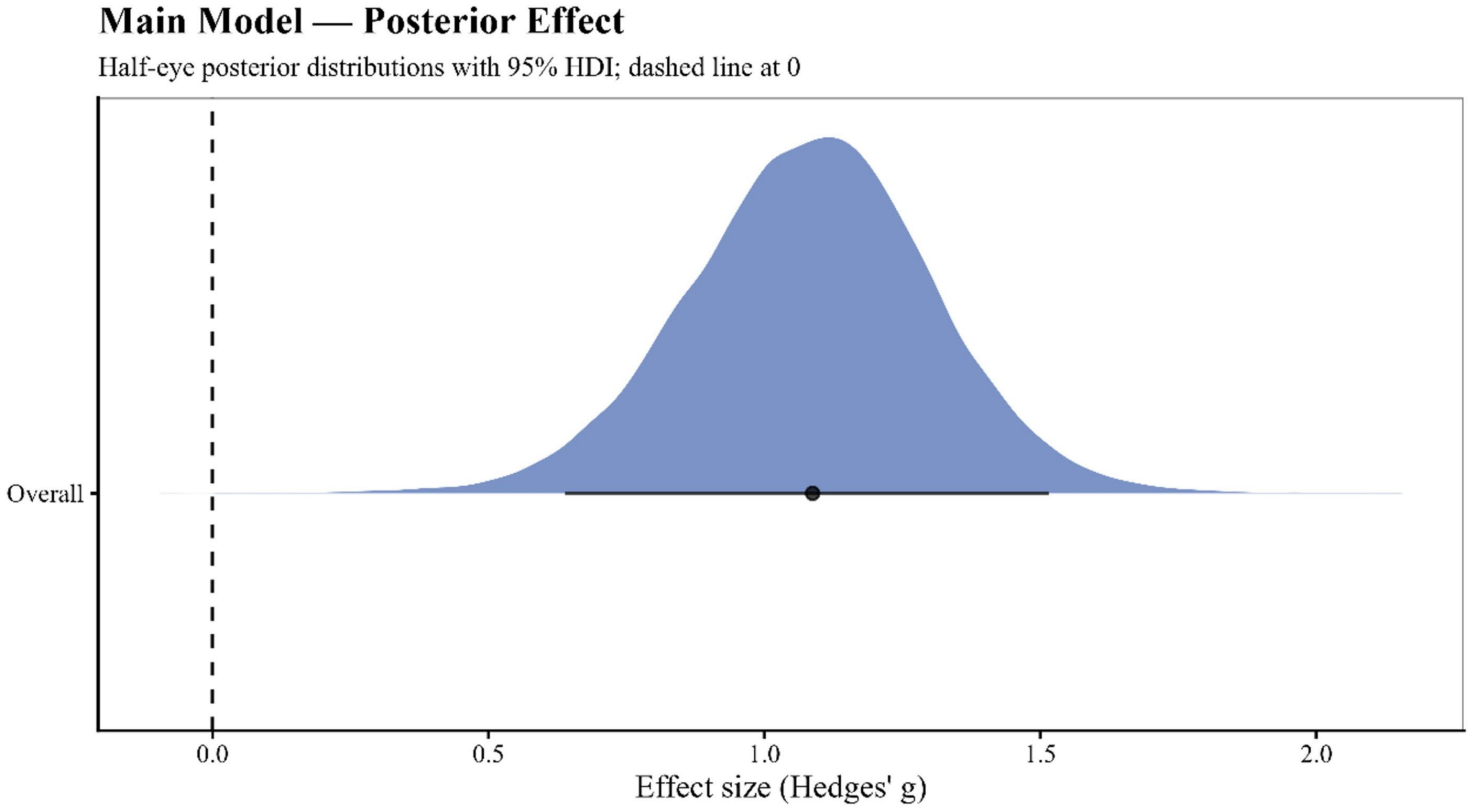
Forest plot for the overall model.

The overall model showed a positive effect of imagery practice across all eligible basketball-related skill and psychological outcomes. The pooled posterior estimate was [*μ*(Hedges’ g): 1.08, 95% HDI: 0.65 to 1.52; BF10: 317.67]. The estimated heterogeneity was tau_within = 0.37 and tau_between = 0.41. Because this model included direct basketball skill outcomes as well as motor-control and self-efficacy outcomes, it should be interpreted as an overall basketball-related outcome estimate rather than as a pure estimate of direct basketball performance. The forest plot for the overall model is presented in Figure 3.

### Outcome subgroup analysis

The outcome subgroup model suggested that effects differed across outcome categories. This model separated direct basketball skill outcomes from secondary motor-control and psychological outcomes. Free throw performance showed a positive effect [*μ*(Hedges’ g): 0.89, 95% HDI: 0.38 to 1.40; BF10: 13.85]. Motor coordination control showed the largest estimate [*μ*(Hedges’ g): 2.14, 95% HDI: 0.89 to 3.28; BF10: 24.04], although this estimate came from a limited evidence base. Self-efficacy also showed a positive estimate [*μ*(Hedges’ g): 1.13, 95% HDI: 0.18 to 2.06; BF10: 3.79].

**Figure 4:**
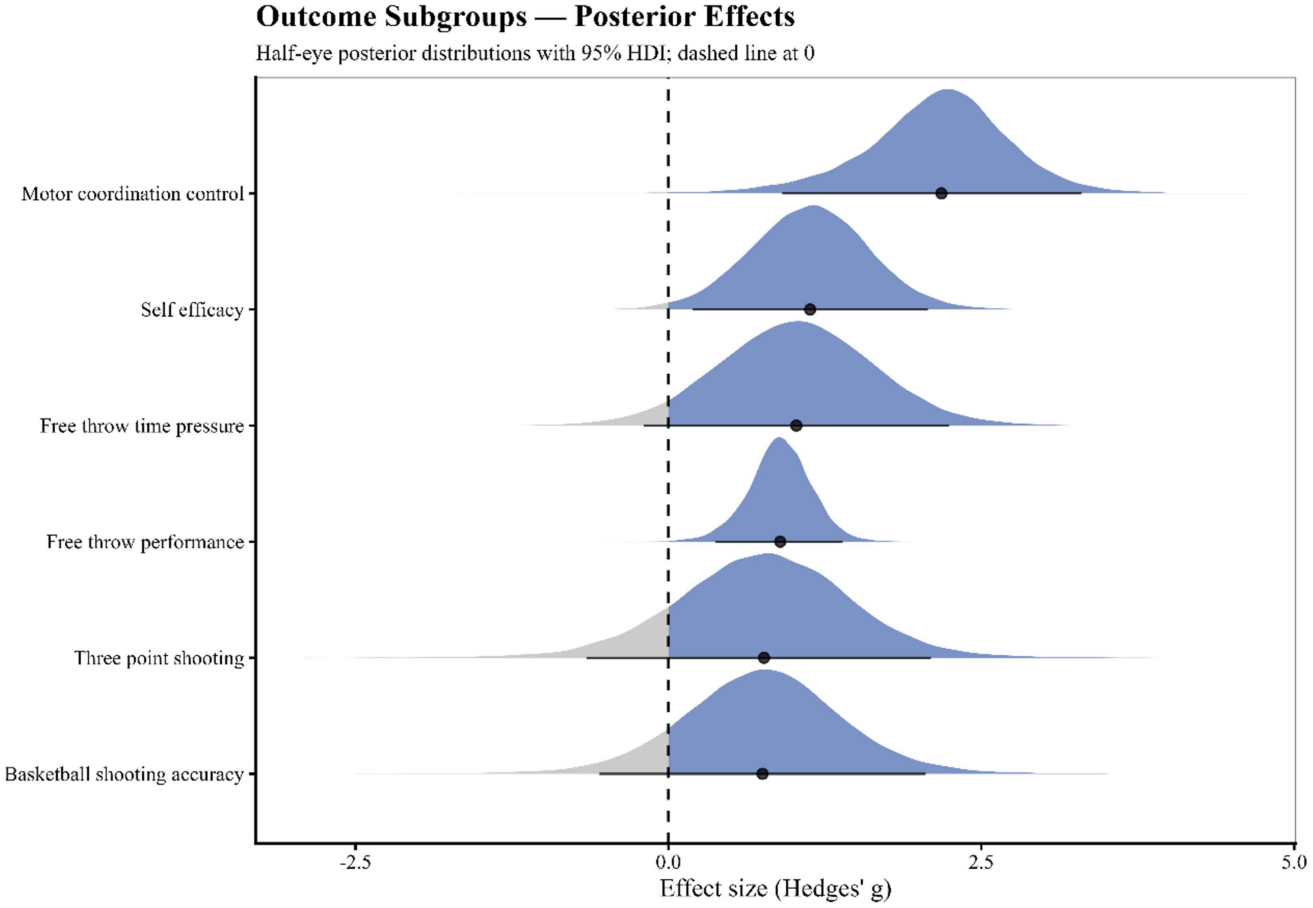
The forest plot in outcome subgroup analysis.

Some outcome estimates were less precise. Basketball shooting accuracy had a wide interval crossing zero [*μ*(Hedges’ g): 0.74, 95% HDI: −0.55 to 2.06; BF10: 0.70]. Free throw performance under time pressure also showed uncertainty [μ(Hedges’ g): 1.02, 95% HDI: −0.21 to 2.23; BF10: 1.25]. Three-point shooting showed a similar pattern [μ(Hedges’ g): 0.75, 95% HDI: −0.71 to 2.04; BF10: 0.66]. The estimated heterogeneity in this model was tau_within = 0.30 and tau_between = 0.31. Accordingly, the positive estimates for motor coordination/control and self-efficacy were considered supportive secondary findings rather than direct evidence of basketball skill improvement. The outcome subgroup forest plot is presented in Figure 4.

### Population subgroup analysis

The exploratory population subgroup model produced the largest posterior mean for novice participants, but this pattern should be interpreted cautiously because subgroup-level evidence was sparse and unevenly distributed. The estimate for novice participants was [*μ*(Hedges’ g): 1.63, 95% HDI: 0.69 to 2.56; BF10: 22.09]. Elite participants showed a favorable direction [*μ*(Hedges’ g): 0.82, 95% HDI: 0.04 to 1.61; BF10: 2.06], although the Bayes factor was weak and the estimate should be regarded as preliminary.

**Figure 5:**
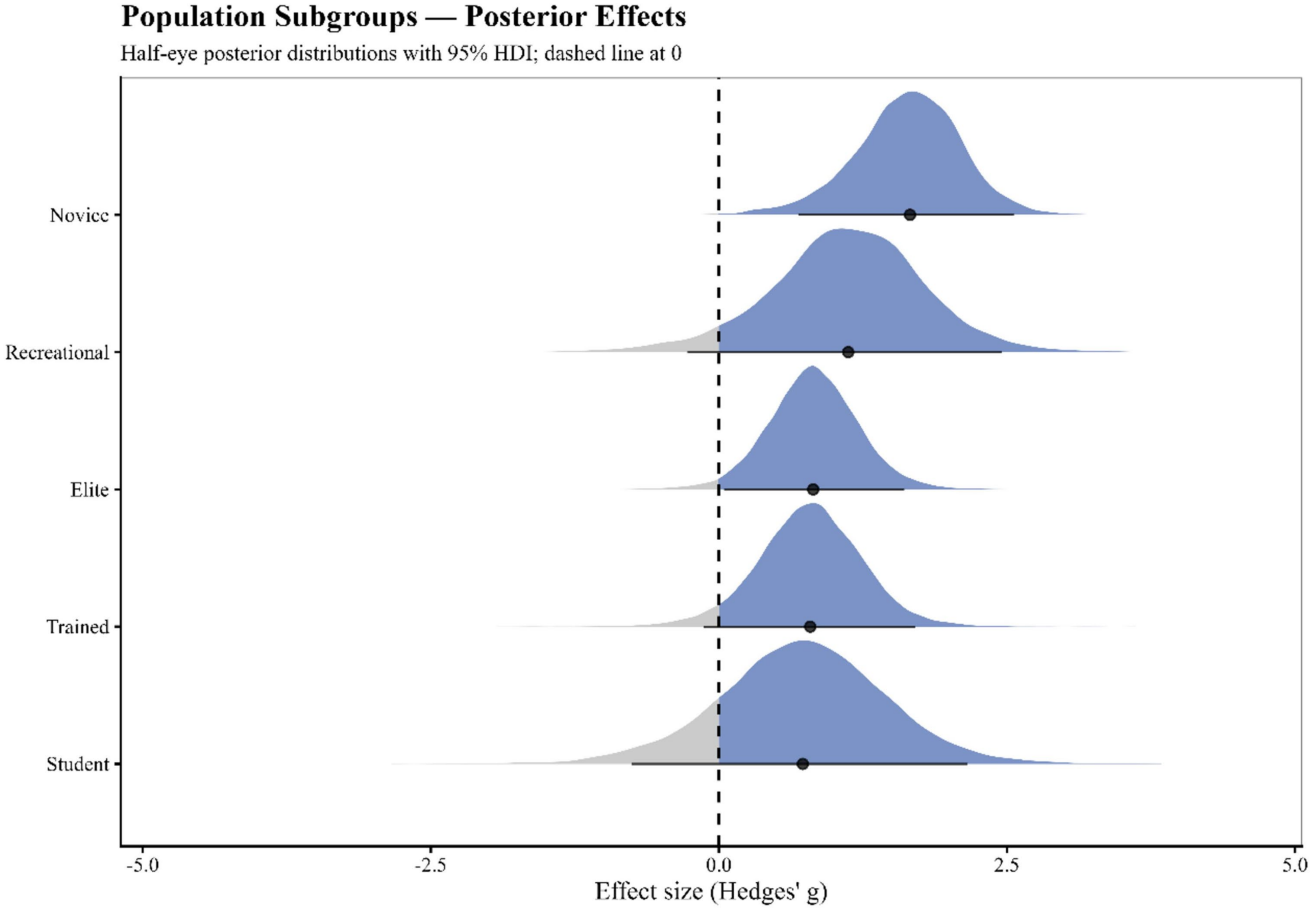
The forest plot in population subgroup analysis.

The estimates for recreational, trained, and student participants were less precise. Recreational participants showed [*μ* (Hedges’ g): 1.10, 95% HDI: −0.28 to 2.45; BF10: 1.53]. Trained participants showed [μ(Hedges’ g): 0.79, 95% HDI: −0.13 to 1.70; BF10: 1.13]. Student participants showed [μ(Hedges’ g): 0.72, 95% HDI: −0.75 to 2.16; BF10: 0.65]. The estimated heterogeneity was tau_within = 0.37 and tau_between = 0.41. These subgroup estimates should be interpreted as exploratory, hypothesis-generating patterns only because some populations were represented by few study units or effect estimates. The model should not be used to make firm conclusions about differential effects between novice, elite, recreational, trained, or student populations. The population subgroup forest plot is presented in Figure 5.

### Moderation analysis

**Figure 6:**
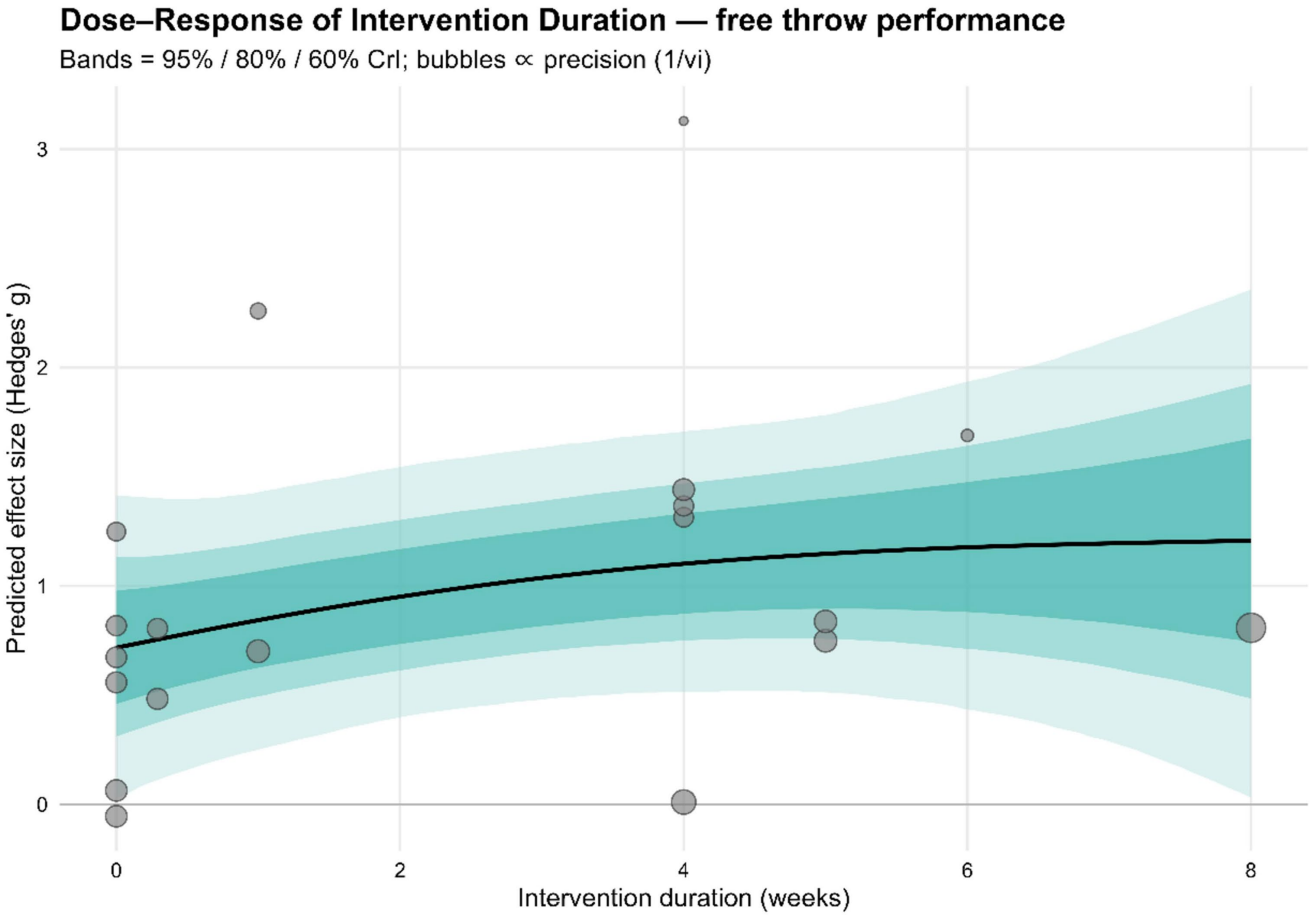
The moderation analysis by duration (weeks).

The dose–response model for free throw performance suggested a gradual increase in the predicted effect as intervention duration increased. The predicted effect was 0.72 at 0 weeks (95% HDI: 0.03 to 1.41), 1.10 at approximately 4 weeks (95% HDI: 0.51 to 1.70), and 1.21 at 8 weeks (95% HDI: 0.03 to 2.36). The uncertainty bands widened at longer durations, indicating lower precision at the upper end of the duration range. Subgroup-specific GAMs were not fitted for other outcomes because the number of effects or the number of unique durations was insufficient. The dose–response plot is presented in Figure 6.

### Quality grade in each outcome

The certainty of evidence was summarized using the GRADE approach. Complete summary-of-findings tables for all key outcomes, including judgments for risk of bias, inconsistency, indirectness, imprecision, and publication bias, are provided in Supplementary File S6.

**Figure 7:**
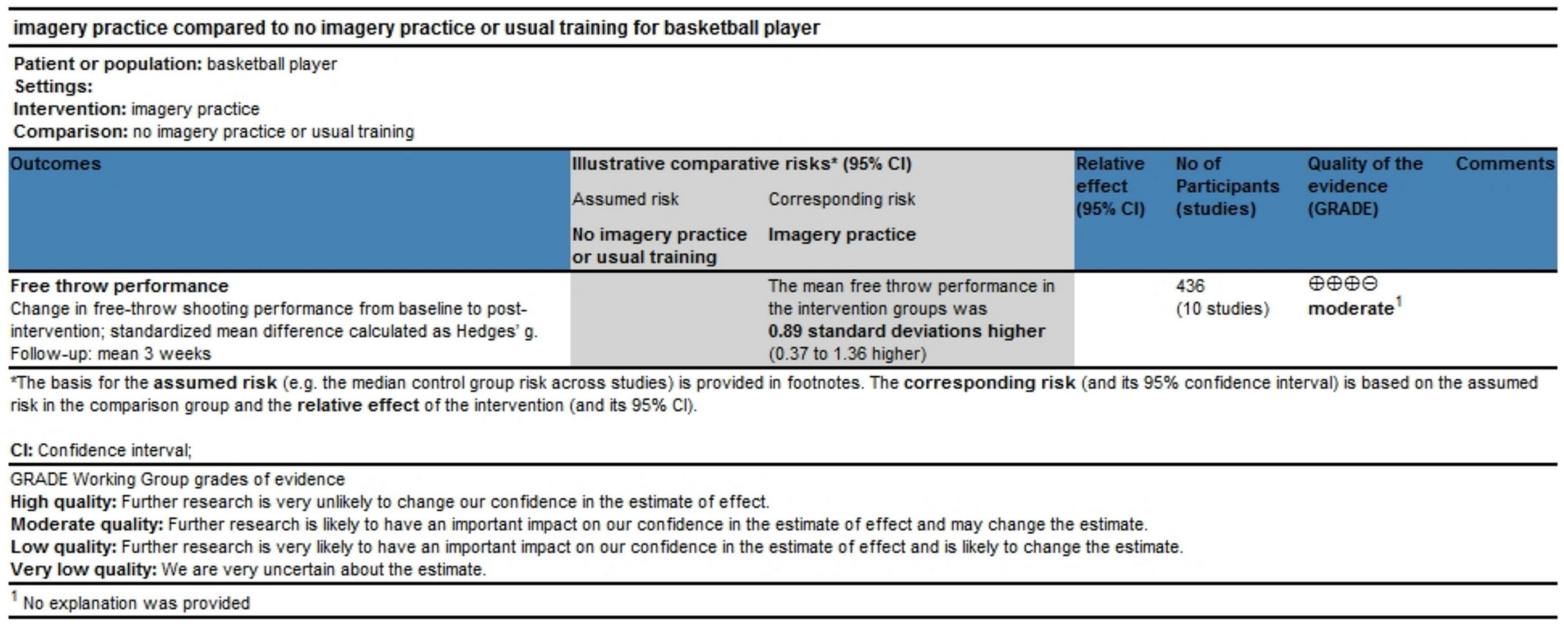
Quality grade of free-throw performance.

For free throw performance, the evidence covered 10 study units and 19 effect estimates, with 436 participant observations contributing to the analyzed contrasts. The posterior estimate favored imagery practice [*μ* (Hedges’ g): 0.89, 95% HDI: 0.38 to 1.40; BF10: 13.85]. The certainty of evidence was rated as low because of concerns about risk of bias and possible small-study effects or publication bias. No serious concern was judged for indirectness because free throw performance was a direct basketball-specific skill outcome, and no serious concern was judged for imprecision because the 95% HDI did not cross zero (see Figure 7).

### Publication bias

**Figure 8:**
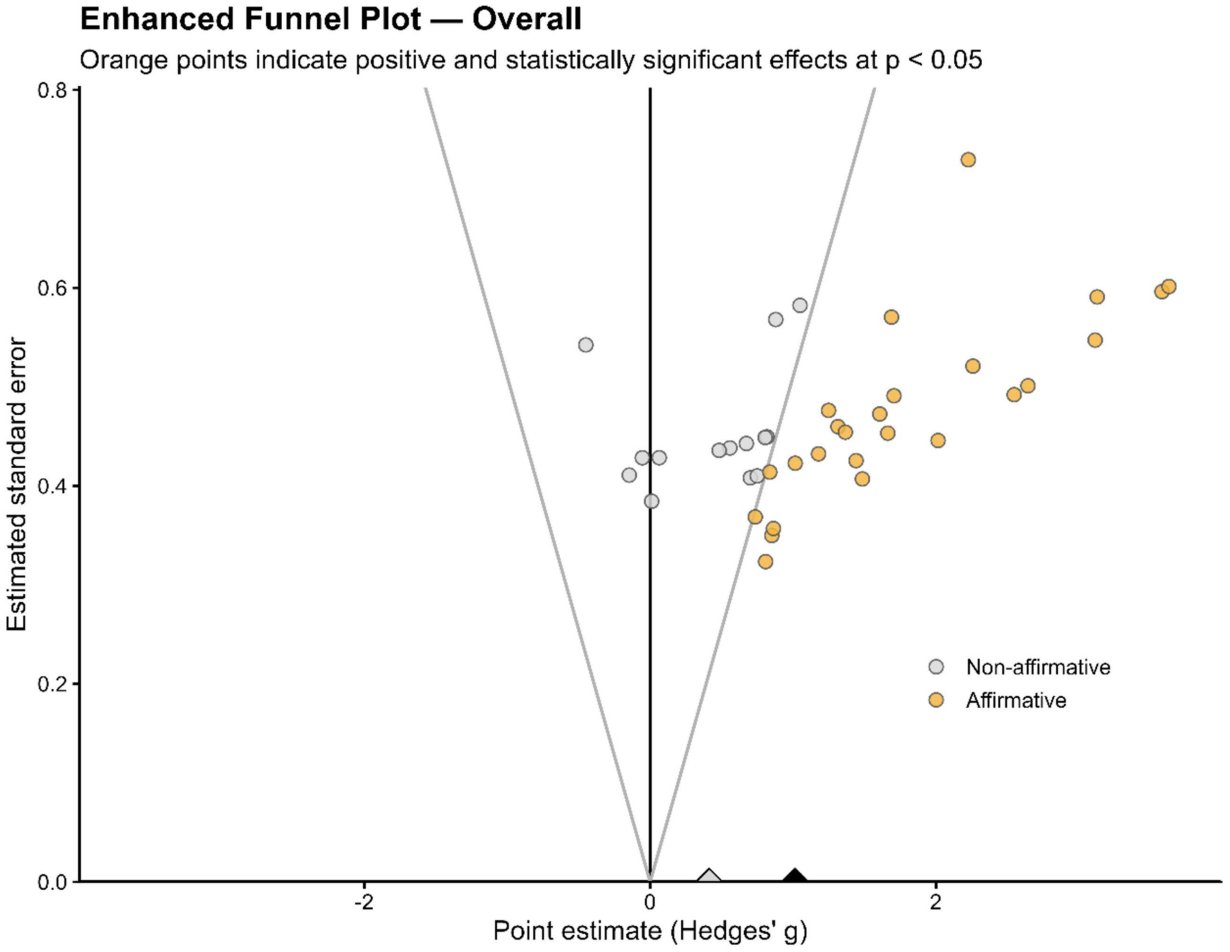
Publication bias funnel plot.

The enhanced funnel plot showed an asymmetric pattern. Among the 39 effects, 25 were classified as affirmative effects and 14 as non-affirmative effects. Affirmative effects were concentrated on the positive side of the plot. The Egger-type regression also suggested a small-study pattern. The standard-error term was positively associated with effect size (*β* = 11.16, *p* < 0.001), and the intercept was statistically different from zero (*β* = −3.98, *p* < 0.001). These findings indicate possible small-study effects or selective evidence patterns. However, the Egger-type result should be interpreted as exploratory because several studies contributed dependent effect sizes. In this context, the effective independent information is reduced, and conventional Egger regression may have reduced statistical power and unstable error-rate properties. The enhanced funnel plot is presented in Figure 8. The numerical Egger-type regression coefficients and publication-bias diagnostics are provided in Supplementary File S5.

## Discussion

This Bayesian multilevel meta-analysis examined the effects of imagery practice on basketball-related skills and psychological outcomes. This systematic review and meta-analysis synthesised evidence on the effects of imagery practice, either delivered alone or combined with usual basketball training, compared with no imagery practice or usual training. It also explored whether intervention dosage, including training duration, was associated with changes in basketball-related performance. Given that the available evidence was most concentrated for free throw performance, this outcome was considered a key domain for certainty assessment.

### Summary of finding

The most consistent direct basketball skill evidence was concentrated in free throw performance. The Bayesian analyses suggested a positive overall pattern across basketball-related outcomes, but the strength and precision of evidence differed by outcome type. The GRADE assessment indicated that the certainty of evidence for free throw performance was low, mainly because of concerns about risk of bias and possible small-study effects or publication bias. In contrast, indirectness and imprecision were not judged as major concerns for this outcome because free throw performance was a direct basketball-specific skill outcome and the 95% HDI did not cross zero.

Compared with prior sport imagery and mental-practice meta-analyses, the present review reached a broadly consistent directional conclusion that imagery practice may be beneficial, but it provides a narrower and more task-specific interpretation. Prior syntheses mainly estimated average effects across multiple sports, task types, and outcome families. The present analysis focused specifically on basketball, distinguished direct basketball skill outcomes from secondary motor-control and psychological outcomes and identified free throw performance as the most defensible basketball-specific outcome in the current evidence base. The Bayesian multilevel approach also adds a methodological contribution by modelling the clustering of 39 effect sizes within 14 study units, rather than treating all effects as independent. This contribution should be understood as an uncertainty-aware synthesis of a small and dependent evidence base, not as evidence that basketball imagery effects are necessarily larger than those reported in broader sport imagery meta-analyses. These findings support imagery practice as a possible adjunct strategy in basketball training, but the strength of this conclusion is limited by risk of bias, small samples, and possible small-study effects. The theoretical and methodological contribution is therefore the use of Bayesian multilevel modelling to address non-independence from multiple outcomes, arms, or testing conditions within the same research source while preserving outcome-level basketball information.

### Interpretation of outcome-specific effects

Free throw performance appeared to be the most interpretable basketball-specific outcome in the current evidence base. This may be partly because free throw is a relatively self-paced, stable, and repeatable motor task. This task structure may allow athletes to rehearse stance, breathing, gaze control, release rhythm, and the expected shot trajectory with relatively consistent demands, which is compatible with evidence on motor imagery, pre-performance routines, and quiet-eye control in free throw shooting (Ktistakis et al., 2026). Imagery practice can be understood as mental rehearsal of voluntary movement without overt execution, and its potential effects are often explained through partial overlap between imagined and executed movement processes (Morone et al., 2022; Egger et al., 1997). Imagery practice can be understood as mental rehearsal of voluntary movement without overt execution, and its potential effects are often explained through partial overlap between imagined and executed movement processes (Rodgers and Pustejovsky, 2021). Therefore, the relatively clear pattern for free throw performance is compatible with the view that imagery practice may be more useful for stable, well-structured motor routines than for less predictable game-like actions. However, this interpretation remains inferential and should not be treated as direct evidence for a specific causal mechanism. This interpretation links the clearer free throw pattern to movement representation, neuromuscular preparation, pre-performance routine stability, and quiet-eye related target fixation; however, it remains a mechanism-based explanation rather than direct causal evidence.

Other basketball-related performance outcomes were less certain. Basketball shooting accuracy, three-point shooting, and free throw performance under time pressure showed wider uncertainty and may involve greater task complexity than standard free throw shooting. This interpretation is consistent with evidence that basketball shooting performance can be affected by shooting distance, defensive pressure, fatigue, and visual-attentional demands (Li et al., 2025; Vencúrik et al., 2022; Vickers et al., 2019; Egger et al., 1997; Rodgers and Pustejovsky, 2021; Ktistakis et al., 2026). In addition, time pressure may influence decision-making and visual search behavior in basketball players, which could make transfer to game-like execution more variable (Goldschmied et al., 2022; Guo and Wang, 2025). Previous work on imagery has also suggested that intervention effects may vary according to delivery format, task characteristics, and implementation details (Toth et al., 2020; Fazel et al., 2018). Some outcomes were represented by limited evidence, which reduced the precision of the estimates. Therefore, the absence of clear effects for these outcomes should be interpreted as insufficient or imprecise evidence, rather than as firm evidence that imagery practice has no effect. Three-point and time-pressured tasks may require greater force scaling, lower-limb and trunk coordination, rapid visual search, and shorter quiet-eye preparation, which may make transfer from imagery scripts more variable unless perceptual, temporal, and pressure constraints are explicitly rehearsed.

Beyond basketball-specific performance, some related outcomes also showed favorable patterns. Motor coordination/control appeared to improve, but this result was based on limited study sources and should be interpreted cautiously. It should not be used as direct evidence that imagery practice improves actual basketball game performance. Self-efficacy also showed a positive pattern, which may reflect a related psychological response to imagery practice rather than a direct basketball performance outcome. This interpretation is consistent with basketball imagery research that assessed both free-throw performance and free-throw self-efficacy, as well as broader evidence linking pre-event self-efficacy with sport performance (Fazel et al., 2018). This pattern should nevertheless be interpreted as indirect psychological evidence rather than direct basketball skill evidence (Fazel et al., 2018). However, self-efficacy is a psychological outcome and should be distinguished from direct basketball skill outcomes such as free throw performance or shooting accuracy. Future studies should jointly assess psychological mechanisms and basketball-specific skill outcomes to clarify whether changes in self-efficacy, attentional control, or pre-performance preparation mediate performance changes.

### Potential moderators and intervention implementation

The population subgroup findings provide only exploratory evidence about possible skill-level differences. The novice subgroup had the largest posterior mean and the elite subgroup showed a favorable direction, but these patterns were based on limited and uneven subgroup evidence. Therefore, they should be treated as hypothesis-generating rather than confirmatory. One possible explanation is that imagery could support developing movement representations in less experienced players or routine stabilization in more experienced players. However, the current subgroup data cannot test these mechanisms directly, and these explanations should be regarded as plausible hypotheses for future research. These explanations are consistent with broader imagery frameworks such as PETTLEP, which emphasize task, timing, learning, emotion, and perspective as important features of imagery design (Morone et al., 2022; Scott et al., 2022). Accordingly, these subgroup patterns should be interpreted only as exploratory and should not be used to infer that novice or elite players benefit more than other groups. The included studies differed in training background, intervention content, outcome measurement, and methodological quality.

The dose–response analysis provides only preliminary guidance for practice. Only the overall evidence base and the free throw performance evidence were suitable for exploratory modelling, while other outcomes were too sparse for meaningful dose–response evaluation. The current evidence is therefore insufficient to identify an optimal intervention period, weekly frequency, or session duration. It only suggests that dose and delivery may be relevant implementation factors. Future trials should report session duration, weekly frequency, total imagery exposure, imagery script content, supervision, adherence, imagery ability, and whether imagery was combined with physical practice. More standardized reporting would improve the reliability of future dose–response and moderator analyses. As cautious operational guidance, coaches may consider 10–15 min imagery sessions, two to three times per week, across approximately 4–8 weeks, while monitoring athlete engagement, imagery ability, and transfer to on-court execution.

### Non-linear dose–response modelling

The use of GAMs was a methodological strength because it allowed intervention duration to be examined without imposing a strictly linear dose–response assumption. This is relevant because imagery practice effects may increase, plateau, or become more variable as training continues. The low-complexity smooth term reduced overfitting risk in a small evidence base with limited unique duration values, so the GAM analysis should be interpreted as exploratory rather than prescriptive.

### Certainty of evidence and methodological considerations

Although the overall direction of the findings was favorable, the certainty of evidence remains limited by methodological concerns. The GRADE approach evaluates certainty using domains such as risk of bias, inconsistency, indirectness, imprecision, and publication bias. In this review, the evidence for free throw performance was rated as low certainty because of risk-of-bias concerns and possible small-study effects or publication bias. The certainty of evidence for the remaining key outcomes was rated as very low because most were based on few study sources, wide uncertainty intervals, indirect outcome constructs, or publication-bias concerns that could not be reliably excluded. The Bayesian model diagnostics suggested acceptable convergence, and the multilevel structure was useful for retaining dependent effects without treating them as independent. However, statistical convergence cannot resolve limitations in the original randomized trials. Relevant trial-level concerns include randomization procedures, allocation concealment, blinding, sample size, attrition, and reporting completeness; these are recognized sources of uncertainty when rating evidence certainty. Publication-bias diagnostics also indicated possible small-study effects or funnel asymmetry. Because funnel plot asymmetry can arise from several causes, including publication bias, heterogeneity, or methodological differences, this finding should be interpreted cautiously (Sterne et al., 2011). In addition, time pressure may influence decision-making and visual search behavior in basketball players, which could make transfer to game-like execution more variable (Goldschmied et al., 2022; Vencúrik et al., 2022). Previous work on imagery has also suggested that intervention effects may vary according to delivery format, task characteristics, and implementation details (Toth et al., 2020; Norouzi et al., 2025). Some outcomes were represented by limited evidence, which reduced the precision of the estimates. Therefore, the absence of clear effects for these outcomes should be interpreted as insufficient or imprecise evidence, rather than as firm evidence that imagery practice has no effect. Three-point and time-pressured tasks may require greater force scaling, lower-limb and trunk coordination, rapid visual search, and shorter quiet-eye preparation, which may make transfer from imagery scripts more variable unless perceptual, temporal, and pressure constraints are explicitly rehearsed.

Beyond basketball-specific performance, some related outcomes also showed favorable patterns. Motor coordination/control appeared to improve, but this result was based on limited study sources and should be interpreted cautiously. It should not be used as direct evidence that imagery practice improves actual basketball game performance. Self-efficacy also showed a positive pattern, which may reflect a related psychological response to imagery practice rather than a direct basketball performance outcome. This interpretation is consistent with basketball imagery research that assessed both free-throw performance and free-throw self-efficacy, as well as broader evidence linking pre-event self-efficacy with sport performance (Norouzi et al., 2025). This pattern should nevertheless be interpreted as indirect psychological evidence rather than direct basketball skill evidence (Norouzi et al., 2025). However, self-efficacy is a psychological outcome and should be distinguished from direct basketball skill outcomes such as free throw performance or shooting accuracy. Future studies should jointly assess psychological mechanisms and basketball-specific skill outcomes to clarify whether changes in self-efficacy, attentional control, or pre-performance preparation mediate performance changes.

### Potential moderators and intervention implementation

The population subgroup findings provide only exploratory evidence about possible skill-level differences. The novice subgroup had the largest posterior mean and the elite subgroup showed a favorable direction, but these patterns were based on limited and uneven subgroup evidence. Therefore, they should be treated as hypothesis-generating rather than confirmatory. One possible explanation is that imagery could support developing movement representations in less experienced players or routine stabilization in more experienced players. However, the current subgroup data cannot test these mechanisms directly, and these explanations should be regarded as plausible hypotheses for future research. These explanations are consistent with broader imagery frameworks such as PETTLEP, which emphasize task, timing, learning, emotion, and perspective as important features of imagery design (Rodgers and Pustejovsky, 2021; Vickers et al., 2019). Accordingly, these subgroup patterns should be interpreted only as exploratory and should not be used to infer that novice or elite players benefit more than other groups. The included studies differed in training background, intervention content, outcome measurement, and methodological quality.

The dose–response analysis provides only preliminary guidance for practice. Only the overall evidence base and the free throw performance evidence were suitable for exploratory modelling, while other outcomes were too sparse for meaningful dose–response evaluation. The current evidence is therefore insufficient to identify an optimal intervention period, weekly frequency, or session duration. It only suggests that dose and delivery may be relevant implementation factors. Future trials should report session duration, weekly frequency, total imagery exposure, imagery script content, supervision, adherence, imagery ability, and whether imagery was combined with physical practice. More standardized reporting would improve the reliability of future dose–response and moderator analyses. As cautious operational guidance, coaches may consider 10–15 min imagery sessions, two to three times per week, across approximately 4–8 weeks, while monitoring athlete engagement, imagery ability, and transfer to on-court execution.

### Non-linear dose–response modelling

The use of GAMs was a methodological strength because it allowed intervention duration to be examined without imposing a strictly linear dose–response assumption. This is relevant because imagery practice effects may increase, plateau, or become more variable as training continues. The low-complexity smooth term reduced overfitting risk in a small evidence base with limited unique duration values, so the GAM analysis should be interpreted as exploratory rather than prescriptive.

### Certainty of evidence and methodological considerations

Although the overall direction of the findings was favorable, the certainty of evidence remains limited by methodological concerns. The GRADE approach evaluates certainty using domains such as risk of bias, inconsistency, indirectness, imprecision, and publication bias. In this review, the evidence for free throw performance was rated as low certainty because of risk-of-bias concerns and possible small-study effects or publication bias. The certainty of evidence for the remaining key outcomes was rated as very low because most were based on few study sources, wide uncertainty intervals, indirect outcome constructs, or publication-bias concerns that could not be reliably excluded. The Bayesian model diagnostics suggested acceptable convergence, and the multilevel structure was useful for retaining dependent effects without treating them as independent. However, statistical convergence cannot resolve limitations in the original randomized trials. Relevant trial-level concerns include randomization procedures, allocation concealment, blinding, sample size, attrition, and reporting completeness; these are recognized sources of uncertainty when rating evidence certainty. Publication-bias diagnostics also indicated possible small-study effects or funnel asymmetry. Because funnel plot asymmetry can arise from several causes, including publication bias, heterogeneity, or methodological differences, this finding should be interpreted cautiously (Guo and Wang, 2025). In addition, conventional Egger regression may have reduced statistical power when multiple dependent effects are extracted from the same study, because the nominal number of effect sizes overstates the effective amount of independent information. Therefore, the publication-bias assessment was used as a sensitivity-oriented diagnostic rather than as definitive evidence of publication bias.

These methodological issues should temper the strength of the conclusions. A positive pooled estimate should not be interpreted as uniform support across all basketball skills. The evidence appears most credible for free throw performance, where the task is more stable and the available evidence is comparatively more interpretable. For more complex or game-like basketball outcomes, the evidence remains limited and imprecise. The overall conclusion should therefore be framed as promising, but not definitive.

### Practical implications and future directions

Imagery practice may be considered as an adjunct strategy in basketball training, especially for free throws, fixed shooting routines, pre-performance routines, and psychological preparation. This interpretation is also consistent with recent sport-imagery intervention studies outside basketball. PETTLEP imagery has shown promise for football passing skill acquisition in novice athletes, suggesting that structured imagery may support motor learning when imagery content is closely matched to the task demands. Functional imagery training has also been examined in elite handball players, where imagery-based work was linked to motor performance and well-being outcomes. These findings broaden the theoretical context of imagery-based interventions in sport, but they should be treated as contextual evidence rather than direct evidence for basketball performance (Norouzi et al., 2025; Norouzi et al., 2019). This practical interpretation is consistent with the broader sport imagery literature, which suggests that imagery interventions can support performance when they are structured, repeated, and matched to task demands (Scott et al., 2022). This practical interpretation is consistent with the broader sport imagery literature, which suggests that imagery interventions can support performance when they are structured, repeated, and matched to task demands (Vickers et al., 2019). Possible applications for novice and elite players should be considered tentative. The current evidence allows only the hypothesis that imagery may support action representation in less experienced players or routine refinement in more experienced players; this requires confirmation in adequately powered subgroup-specific trials. However, imagery practice should be used as a supplement to technical practice on court. Practitioners should adapt imagery content according to athlete level, skill type, imagery ability, and training goals. Practical scripts should be differentiated by task and athlete level: free throw scripts should emphasize stance, breathing, quiet-eye target fixation, release rhythm, and expected shot trajectory; game-like shooting scripts should include distance, defensive or time constraints, decision cues, and recovery after misses; novice scripts may emphasize basic movement representation, whereas elite scripts may emphasize routine refinement and pressure regulation.

The applied contribution of this review is the sport-specific separation of basketball skill outcomes. Treating self-paced free throws separately from open-skill, distance-based, or pressure-sensitive shooting outcomes improves ecological interpretation and coaching transferability.

This review has several limitations. Some outcomes included only a small amount of evidence, which reduced precision. Several studies had methodological concerns related to randomization, allocation concealment, blinding, sample size, or reporting completeness. Intervention protocols also varied in imagery script, duration, supervision, and combination with physical training. Therefore, effects from combined protocols should be interpreted as imagery-containing package effects when co-interventions were not fully matched. Outcome measures were heterogeneous, and some tests may have been only indirectly related to actual game performance. Possible small-study effects may also have influenced the overall estimate. Future research should use larger samples, preregistered protocols, standardized imagery interventions, longer follow-up, game-relevant outcomes, and mechanism measures. Future studies should also examine whether the effects of imagery practice differ by skill complexity, training level, imagery ability, and intervention dose. Future studies should use preregistered and adequately powered randomized controlled trials with standardized imagery practice protocols. Protocols should report imagery perspective, sensory modality, script content, session duration, weekly frequency, supervision, adherence, imagery ability, and concurrent physical practice. Mechanism-focused trials should include eye-tracking, quiet-eye duration, gaze fixation stability, electromyography, EEG or ERP indices, and psychological mediators. Combined imagery-video observation protocols and interactions between imagery practice and psychological skill training should also be tested using designs that can separate additive and interactive effects.

Taken together, imagery practice was associated with favorable changes in basketball-related performance, but the strength of the evidence differed across outcomes. Free throw performance currently provides the clearest and most interpretable evidence. Evidence for more complex or game-like basketball skills remains limited and imprecise. Imagery practice may therefore be considered as a supplementary training strategy, while higher-quality studies are still needed to clarify the skills, populations, and training conditions for which they are most applicable. The Bayesian multilevel and GAM analyses provide a more uncertainty-aware framework for synthesizing clustered basketball imagery evidence and exploring possible non-linear dose–response patterns.

## Conclusion

Imagery practice appears to be associated with improvements in basketball-related outcomes. The most consistent direct basketball skill evidence was observed for free throw performance, which may reflect the stable, self-paced, and repeatable nature of this skill. However, the GRADE certainty for free throw performance was low because of risk-of-bias concerns and possible small-study effects. Evidence for more complex or game-like basketball skills, such as three-point shooting, general shooting accuracy, and free throws under time pressure, remains very uncertain because the certainty of evidence was very low. Motor coordination/control and self-efficacy findings should be interpreted as secondary basketball-related outcomes rather than direct evidence of basketball skill improvement, and their certainty of evidence was also very low. These findings suggest that imagery practice may be considered as an adjunct to regular basketball training, especially for fixed skill routines and pre-performance preparation. Future high-quality studies are needed to clarify which basketball skills, athlete populations, and intervention doses are most likely to benefit from imagery practice. Future high-quality studies should also clarify whether quiet-eye related preparation, neuromuscular control, and pre-performance routine stability mediate improvements in specific basketball skills.

## Data Availability

The original contributions presented in the study are included in the article/Supplementary material, further inquiries can be directed to the corresponding author.
