# Supplementary material for: Effects of imagery practice on basketball-related skills and psychological outcomes: a systematic review and Bayesian meta-analysis
Source: Front Psychol. 2026 Jul 31;17:1888190. doi: 10.3389/fpsyg.2026.1888190 (PMC13472922; doi:10.3389/fpsyg.2026.1888190)
Supplement: Supplementary file 1 [file Supplementary_file_1.docx]

| **Section and Topic** | **Item #** | **Checklist item** | **Location where item is reported** |
| --- | --- | --- | --- |
| **TITLE** | | | **Section heading; item locations listed below.** |
| Title | 1 | Identify the report as a systematic review. | Title page: 'Effects of Imagery Practice on Basketball-Related Skills and Psychological Outcomes: A Systematic Review and Bayesian Meta-Analysis'. |
| **ABSTRACT** | | | **Section heading; item locations listed below.** |
| Abstract | 2 | See the PRISMA 2020 for Abstracts checklist. | Abstract: Background, Objective, Methods, Results, and Conclusion. |
| **INTRODUCTION** | | | **Section heading; item locations listed below.** |
| Rationale | 3 | Describe the rationale for the review in the context of existing knowledge. | Introduction, paragraphs 1-4. |
| Objectives | 4 | Provide an explicit statement of the objective(s) or question(s) the review addresses. | Introduction, final paragraph. |
| **METHODS** | | | **Section heading; item locations listed below.** |
| Eligibility criteria | 5 | Specify the inclusion and exclusion criteria for the review and how studies were grouped for the syntheses. | Methods: Eligibility Criteria. |
| Information sources | 6 | Specify all databases, registers, websites, organisations, reference lists and other sources searched or consulted to identify studies. Specify the date when each source was last searched or consulted. | Methods: Information Sources. |
| Search strategy | 7 | Present the full search strategies for all databases, registers and websites, including any filters and limits used. | Supplementary File S2: complete search strategies for PubMed, Web of Science, Scopus, and EBSCO. |
| Selection process | 8 | Specify the methods used to decide whether a study met the inclusion criteria of the review, including how many reviewers screened each record and each report retrieved, whether they worked independently, and if applicable, details of automation tools used in the process. | Methods: Study Selection and Data Collection. |
| Data collection process | 9 | Specify the methods used to collect data from reports, including how many reviewers collected data from each report, whether they worked independently, any processes for obtaining or confirming data from study investigators, and if applicable, details of automation tools used in the process. | Methods: Study Selection and Data Collection. |
| Data items | 10a | List and define all outcomes for which data were sought. Specify whether all results that were compatible with each outcome domain in each study were sought (e.g. for all measures, time points, analyses), and if not, the methods used to decide which results to collect. | Methods: Eligibility Criteria; Methods: Data Items. |
|  | 10b | List and define all other variables for which data were sought (e.g. participant and intervention characteristics, funding sources). Describe any assumptions made about any missing or unclear information. | Methods: Study Selection and Data Collection; Methods: Data Items. |
| Study risk of bias assessment | 11 | Specify the methods used to assess risk of bias in the included studies, including details of the tool(s) used, how many reviewers assessed each study and whether they worked independently, and if applicable, details of automation tools used in the process. | Methods: Risk of Bias Assessment; Supplementary File S4. |
| Effect measures | 12 | Specify for each outcome the effect measure(s) (e.g. risk ratio, mean difference) used in the synthesis or presentation of results. | Methods: Data Items; Methods: Summary Measures and Synthesis. |
| Synthesis methods | 13a | Describe the processes used to decide which studies were eligible for each synthesis (e.g. tabulating the study intervention characteristics and comparing against the planned groups for each synthesis (item #5)). | Methods: Eligibility Criteria; Methods: Summary Measures and Synthesis; Methods: Moderation Analysis. |
|  | 13b | Describe any methods required to prepare the data for presentation or synthesis, such as handling of missing summary statistics, or data conversions. | Methods: Data Items; Methods: Summary Measures and Synthesis. |
|  | 13c | Describe any methods used to tabulate or visually display results of individual studies and syntheses. | Methods: Summary Measures and Synthesis; Results: The Results of Meta-analysis; Figures 3-8; Supplementary Files S3-S5. |
|  | 13d | Describe any methods used to synthesize results and provide a rationale for the choice(s). If meta-analysis was performed, describe the model(s), method(s) to identify the presence and extent of statistical heterogeneity, and software package(s) used. | Methods: Summary Measures and Synthesis. |
|  | 13e | Describe any methods used to explore possible causes of heterogeneity among study results (e.g. subgroup analysis, meta-regression). | Methods: Moderation Analysis. |
|  | 13f | Describe any sensitivity analyses conducted to assess robustness of the synthesized results. | Not applicable: no formal sensitivity analysis was conducted. |
| Reporting bias assessment | 14 | Describe any methods used to assess risk of bias due to missing results in a synthesis (arising from reporting biases). | Methods: Summary Measures and Synthesis, publication-bias methods; Results: Publication Bias; Supplementary File S5. |
| Certainty assessment | 15 | Describe any methods used to assess certainty (or confidence) in the body of evidence for an outcome. | Methods: Certainty in Evidence. |
| **RESULTS** | | | **Section heading; item locations listed below.** |
| Study selection | 16a | Describe the results of the search and selection process, from the number of records identified in the search to the number of studies included in the review, ideally using a flow diagram. | Results: Study Selection; Figure 1. |
|  | 16b | Cite studies that might appear to meet the inclusion criteria, but which were excluded, and explain why they were excluded. | Results: Study Selection; full-text exclusion reasons provided. |
| Study characteristics | 17 | Cite each included study and present its characteristics. | Results: Characteristics of Included Studies; Supplementary File S3. |
| Risk of bias in studies | 18 | Present assessments of risk of bias for each included study. | Results: Quality Assessment; Figure 2; Supplementary File S4. |
| Results of individual studies | 19 | For all outcomes, present, for each study: (a) summary statistics for each group (where appropriate) and (b) an effect estimate and its precision (e.g. confidence/credible interval), ideally using structured tables or plots. | Results: The Results of Meta-analysis; Figures 3-8; Supplementary Files S3 and S5. |
| Results of syntheses | 20a | For each synthesis, briefly summarise the characteristics and risk of bias among contributing studies. | Results: Characteristics of Included Studies; Results: Quality Assessment; Results: The Results of Meta-analysis. |
|  | 20b | Present results of all statistical syntheses conducted. If meta-analysis was done, present for each the summary estimate and its precision (e.g. confidence/credible interval) and measures of statistical heterogeneity. If comparing groups, describe the direction of the effect. | Results: Overall model; Outcome Subgroup Analysis; Population Subgroup Analysis; Moderation Analysis; Figures 3-6. |
|  | 20c | Present results of all investigations of possible causes of heterogeneity among study results. | Results: Outcome Subgroup Analysis; Population Subgroup Analysis; Moderation Analysis; Figures 4-6. |
|  | 20d | Present results of all sensitivity analyses conducted to assess the robustness of the synthesized results. | Not applicable: no sensitivity analyses were conducted. |
| Reporting biases | 21 | Present assessments of risk of bias due to missing results (arising from reporting biases) for each synthesis assessed. | Results: Publication Bias; Figure 8; Supplementary File S5. |
| Certainty of evidence | 22 | Present assessments of certainty (or confidence) in the body of evidence for each outcome assessed. | Results: Quality Grade in Each Outcome; Figure 7; GRADE summary-of-findings files. |
| **DISCUSSION** | | | **Section heading; item locations listed below.** |
| Discussion | 23a | Provide a general interpretation of the results in the context of other evidence. | Discussion: Summary of Finding; Interpretation of Outcome-Specific Effects. |
|  | 23b | Discuss any limitations of the evidence included in the review. | Discussion: Certainty of Evidence and Methodological Considerations; Practical Implications and Future Directions. |
|  | 23c | Discuss any limitations of the review processes used. | Discussion: Certainty of Evidence and Methodological Considerations; Practical Implications and Future Directions. |
|  | 23d | Discuss implications of the results for practice, policy, and future research. | Discussion: Practical Implications and Future Directions; Conclusion. |
| **OTHER INFORMATION** | | | **Section heading; item locations listed below.** |
| Registration and protocol | 24a | Provide registration information for the review, including register name and registration number, or state that the review was not registered. | Methods, first paragraph: PROSPERO registration CRD420261389048. |
|  | 24b | Indicate where the review protocol can be accessed, or state that a protocol was not prepared. | Methods, first paragraph: protocol access indicated through PROSPERO registration CRD420261389048. |
|  | 24c | Describe and explain any amendments to information provided at registration or in the protocol. | Not applicable: no amendments to registration or protocol were reported in the manuscript. |
| Support | 25 | Describe sources of financial or non-financial support for the review, and the role of the funders or sponsors in the review. | Funding section: None. |
| Competing interests | 26 | Declare any competing interests of review authors. | Conflicts of Interest section: authors declare no conflicts of interest. |
| Availability of data, code and other materials | 27 | Report which of the following are publicly available and where they can be found: template data collection forms; data extracted from included studies; data used for all analyses; analytic code; any other materials used in the review. | Supplementary Files S1-S5; Supplementary File S3 contains extracted/effect-size data; Supplementary File S5 contains Egger-test results; result file list identifies analytic outputs. |

*From:*  Page MJ, McKenzie JE, Bossuyt PM, Boutron I, Hoffmann TC, Mulrow CD, et al. The PRISMA 2020 statement: an updated guideline for reporting systematic reviews. BMJ 2021;372:n71. doi: 10.1136/bmj.n71. This work is licensed under CC BY 4.0. To view a copy of this license, visit <https://creativecommons.org/licenses/by/4.0/>
