# Supplementary material for: Effects of imagery practice on basketball-related skills and psychological outcomes: a systematic review and Bayesian meta-analysis
Source: Front Psychol. 2026 Jul 31;17:1888190. doi: 10.3389/fpsyg.2026.1888190 (PMC13472922; doi:10.3389/fpsyg.2026.1888190)
Supplement: Supplementary file 2 [file Supplementary_file_2.docx]

**Supplementary File S2. Complete Search Strategies**

**Search overview**

| **Databases searched** | PubMed, Web of Science, Scopus, and EBSCOhost. |
| --- | --- |
| **Final search date** | 10 April 2026. |
| **Publication-date limits** | No publication-date restriction was applied. All records available in each database up to the final search date were eligible for screening. |
| **Boolean structure** | (basketball terms) AND (imagery, psychological intervention, or mental training terms) AND (performance or skill outcome terms). |
| **Grey-literature sources** | Separate searches of theses or dissertations, conference proceedings, and trial registries were not conducted. This limitation is reported in the manuscript. |

**PubMed**

( "Basketball"[Mesh] OR basketball*[tiab] OR "basketball player*"[tiab] )

AND

( psycholog*[tiab] OR mental[tiab] OR "mental training"[tiab] OR "mental skill*"[tiab] OR "psychological skill*"[tiab] OR "psychological skills training"[tiab] OR "mental skills training"[tiab] OR intervention*[tiab] OR "psychological intervention*"[tiab] OR "mental intervention*"[tiab] OR self-talk[tiab] OR "self talk"[tiab] OR imagery[tiab] OR "mental imagery"[tiab] OR visualization[tiab] OR visualisation[tiab] OR "mental practice"[tiab] OR "mental rehearsal"[tiab] OR mindfulness[tiab] OR meditation[tiab] OR relaxation[tiab] OR "goal setting"[tiab] OR biofeedback[tiab] OR neurofeedback[tiab] OR hypnosis[tiab] OR "attention training"[tiab] OR "concentration training"[tiab] OR "breathing training"[tiab] )

AND

( performance[tiab] OR "sport performance"[tiab] OR "sports performance"[tiab] OR "athletic performance"[tiab] OR "motor performance"[tiab] OR skill*[tiab] OR shooting[tiab] OR accuracy[tiab] OR "free throw*"[tiab] OR "jump shot*"[tiab] OR passing[tiab] OR dribbling[tiab] OR agility[tiab] OR sprint*[tiab] OR endurance[tiab] OR "reaction time"[tiab] OR "decision making"[tiab] )

**Web of Science**

TS=(

(basketball* OR "basketball player" OR "basketball players")

AND

(psycholog* OR mental OR "mental training" OR "mental skill*" OR "psychological skill*" OR "psychological skills training" OR "mental skills training" OR intervention* OR "psychological intervention*" OR "mental intervention*" OR self-talk OR "self talk" OR imagery OR "mental imagery" OR visualization OR visualisation OR "mental practice" OR "mental rehearsal" OR mindfulness OR meditation OR relaxation OR "goal setting" OR biofeedback OR neurofeedback OR hypnosis OR "attention training" OR "concentration training" OR "breathing training")

AND

(performance OR "sport performance" OR "sports performance" OR "athletic performance" OR "motor performance" OR skill* OR shooting OR accuracy OR "free throw*" OR "jump shot*" OR passing OR dribbling OR agility OR sprint* OR endurance OR "reaction time" OR "decision making")

)

**Scopus**

TITLE-ABS-KEY( basketball* OR "basketball player" OR "basketball players" )

AND

TITLE-ABS-KEY( psycholog* OR mental OR "mental training" OR "mental skill*" OR "psychological skill*" OR "psychological skills training" OR "mental skills training" OR intervention* OR "psychological intervention*" OR "mental intervention*" OR self-talk OR "self talk" OR imagery OR "mental imagery" OR visualization OR visualisation OR "mental practice" OR "mental rehearsal" OR mindfulness OR meditation OR relaxation OR "goal setting" OR biofeedback OR neurofeedback OR hypnosis OR "attention training" OR "concentration training" OR "breathing training" )

AND

TITLE-ABS-KEY( performance OR "sport performance" OR "sports performance" OR "athletic performance" OR "motor performance" OR skill* OR shooting OR accuracy OR "free throw*" OR "jump shot*" OR passing OR dribbling OR agility OR sprint* OR endurance OR "reaction time" OR "decision making" )

**EBSCOhost**

S1: (TI basketball* OR AB basketball* OR SU basketball OR TI "basketball player" OR AB "basketball player" OR TI "basketball players" OR AB "basketball players")

S2: (TI psycholog* OR AB psycholog* OR TI mental OR AB mental OR TI "mental training" OR AB "mental training" OR TI "mental skill*" OR AB "mental skill*" OR TI "psychological skill*" OR AB "psychological skill*" OR TI "psychological skills training" OR AB "psychological skills training" OR TI "mental skills training" OR AB "mental skills training" OR TI intervention* OR AB intervention* OR TI "psychological intervention*" OR AB "psychological intervention*" OR TI "mental intervention*" OR AB "mental intervention*" OR TI self-talk OR AB self-talk OR TI "self talk" OR AB "self talk" OR TI imagery OR AB imagery OR TI "mental imagery" OR AB "mental imagery" OR TI visualization OR AB visualization OR TI visualisation OR AB visualisation OR TI "mental practice" OR AB "mental practice" OR TI "mental rehearsal" OR AB "mental rehearsal" OR TI mindfulness OR AB mindfulness OR TI meditation OR AB meditation OR TI relaxation OR AB relaxation OR TI "goal setting" OR AB "goal setting" OR TI biofeedback OR AB biofeedback OR TI neurofeedback OR AB neurofeedback OR TI hypnosis OR AB hypnosis OR TI "attention training" OR AB "attention training" OR TI "concentration training" OR AB "concentration training" OR TI "breathing training" OR AB "breathing training")

S3: (TI performance OR AB performance OR TI "sport performance" OR AB "sport performance" OR TI "sports performance" OR AB "sports performance" OR TI "athletic performance" OR AB "athletic performance" OR TI "motor performance" OR AB "motor performance" OR TI skill* OR AB skill* OR TI shooting OR AB shooting OR TI accuracy OR AB accuracy OR TI "free throw*" OR AB "free throw*" OR TI "jump shot*" OR AB "jump shot*" OR TI passing OR AB passing OR TI dribbling OR AB dribbling OR TI agility OR AB agility OR TI sprint* OR AB sprint* OR TI endurance OR AB endurance OR TI "reaction time" OR AB "reaction time" OR TI "decision making" OR AB "decision making")

Final combination: S1 AND S2 AND S3
