# Supplementary material for: Effects of imagery practice on basketball-related skills and psychological outcomes: a systematic review and Bayesian meta-analysis
Source: Front Psychol. 2026 Jul 31;17:1888190. doi: 10.3389/fpsyg.2026.1888190 (PMC13472922; doi:10.3389/fpsyg.2026.1888190)
Supplement: Supplementary file 3 [file Supplementary_file_6.docx]

Figure S1. Summary of findings (GRADE) for imagery practice versus no imagery practice or usual training on basketball performance outcomes.

**Table S6.1. Summary of Findings for imagery practice versus no imagery practice or usual training on basketball-related skill and psychological outcomes.**

| **Outcome** | **Outcome type** | **Participant observations / study units / effect estimates** | **Posterior effect estimate** | **GRADE certainty** | **Interpretation** |
| --- | --- | --- | --- | --- | --- |
| Free throw performance | Direct basketball skill outcome; critical outcome | 436 participant observations; 10 study units; 19 effect estimates | Hedges' g = 0.89; 95% HDI: 0.38 to 1.40; BF10 = 13.85 | Low | Imagery practice may improve free throw performance, but confidence is limited by risk-of-bias concerns and possible small-study effects or publication bias. |
| Basketball shooting accuracy | Direct basketball skill outcome | 48 participant observations; 1 study unit; 4 effect estimates | Hedges' g = 0.74; 95% HDI: -0.55 to 2.06; BF10 = 0.70 | Very low | The effect is very uncertain because the evidence is sparse and the 95% HDI crosses zero. |
| Free throw performance under time pressure | Direct basketball skill outcome | 48 participant observations; 1 study unit; 2 effect estimates | Hedges' g = 1.02; 95% HDI: -0.21 to 2.23; BF10 = 1.25 | Very low | The effect is very uncertain because the evidence is sparse and the 95% HDI crosses zero. |
| Three-point shooting | Direct basketball skill outcome | 69 participant observations; 1 study unit; 2 effect estimates | Hedges' g = 0.75; 95% HDI: -0.71 to 2.04; BF10 = 0.66 | Very low | The effect is very uncertain because the evidence is sparse and the 95% HDI crosses zero. |
| Motor coordination/control | Secondary basketball-related motor-control outcome | 240 participant observations; 1 study unit; 8 effect estimates | Hedges' g = 2.14; 95% HDI: 0.89 to 3.28; BF10 = 24.04 | Very low | The estimate favors imagery practice, but the evidence is indirect and based on a sparse evidence base. |
| Self-efficacy | Secondary basketball-related psychological outcome | 93 participant observations; 2 study units; 4 effect estimates | Hedges' g = 1.13; 95% HDI: 0.18 to 2.06; BF10 = 3.79 | Very low | The estimate favors imagery practice, but this self-reported psychological outcome should not be interpreted as direct basketball skill evidence. |

Notes. Positive Hedges' g values favor imagery practice. BF10 values were interpreted as supplementary evidence alongside posterior estimates and 95% HDIs. Participant observations refer to contrast-level participant observations contributing to the analyzed effect estimates; because the Bayesian multilevel model retained multiple dependent effects from some study units, these values should not be interpreted as unique participants.

**Table S6.2. Domain-level GRADE judgments for key outcomes.**

| **Outcome** | **Risk of bias** | **Inconsistency** | **Indirectness** | **Imprecision** | **Publication bias** | **Final certainty** |
| --- | --- | --- | --- | --- | --- | --- |
| Free throw performance | Serious concern. Several trials had unclear or insufficient reporting of randomization, allocation concealment, intervention delivery, or selective reporting. | No serious concern. This was the most represented outcome and the direction of evidence was comparatively consistent. | No serious concern. Free throw performance is a direct basketball-specific skill outcome. | No serious concern. The 95% HDI did not cross zero. | Serious concern. Funnel-plot and Egger-type diagnostics suggested possible small-study effects, and grey literature was not searched. | Low |
| Basketball shooting accuracy | Serious concern. Risk-of-bias concerns were present in the included evidence base. | Serious concern. The outcome was based on one study unit, so consistency across independent studies could not be assessed. | No serious concern. The outcome is a direct basketball shooting outcome. | Very serious concern. The evidence was sparse and the 95% HDI crossed zero. | Serious concern. Publication bias could not be excluded in a sparse evidence base, and the overall analysis suggested small-study effects. | Very low |
| Free throw performance under time pressure | Serious concern. Risk-of-bias concerns were present in the included evidence base. | Serious concern. The outcome was based on one study unit, so consistency across independent studies could not be assessed. | No serious concern. The outcome is a direct basketball skill outcome under a pressure-related task condition. | Very serious concern. The evidence was sparse and the 95% HDI crossed zero. | Serious concern. Publication bias could not be excluded in a sparse evidence base, and the overall analysis suggested small-study effects. | Very low |
| Three-point shooting | Serious concern. Risk-of-bias concerns were present in the included evidence base. | Serious concern. The outcome was based on one study unit, so consistency across independent studies could not be assessed. | No serious concern. The outcome is a direct basketball shooting outcome. | Very serious concern. The evidence was sparse and the 95% HDI crossed zero. | Serious concern. Publication bias could not be excluded in a sparse evidence base, and the overall analysis suggested small-study effects. | Very low |
| Motor coordination/control | Serious concern. Risk-of-bias concerns were present in the included evidence base. | Serious concern. The estimate was based on one study unit with multiple dependent effects. | Serious concern. This was a secondary motor-control outcome and should not be interpreted as direct basketball skill performance. | Serious concern. Although the 95% HDI did not cross zero, the evidence was sparse and came from one study unit. | Serious concern. Publication bias could not be excluded in a sparse evidence base, and the overall analysis suggested small-study effects. | Very low |
| Self-efficacy | Serious concern. Self-efficacy was self-reported, and lack of participant blinding may have introduced expectancy or demand effects. | Serious concern. The outcome was based on two study units and four dependent effect estimates. | Serious concern. This was a psychological outcome and should not be interpreted as direct basketball skill performance. | Serious concern. The evidence was sparse and the interval was wide. | Serious concern. Publication bias could not be excluded in a sparse evidence base, and the overall analysis suggested small-study effects. | Very low |

Notes. Randomized trials started as high-certainty evidence. Serious concerns indicate downgrading by one level, and very serious concerns indicate downgrading by two levels. Certainty was not downgraded below very low. GRADE domains were interpreted in relation to each outcome subgroup.
