# Supplementary material for: Effects of imagery practice on basketball-related skills and psychological outcomes: a systematic review and Bayesian meta-analysis
Source: Front Psychol. 2026 Jul 31;17:1888190. doi: 10.3389/fpsyg.2026.1888190 (PMC13472922; doi:10.3389/fpsyg.2026.1888190)
Supplement: Supplementary file 4 [file Supplementary_file_7.docx]

**Supplementary File S7. MCMC Diagnostic Plots and Effective Sample Size Values**

This supplementary file presents Markov chain trace plots, chain-wise posterior density plots, and bulk/tail effective sample size diagnostics for the Bayesian meta-analysis models.

**Figure S7.1.** Main model: Trace plot. MCMC trace plots by Markov chain. Well-mixed chains should overlap without sustained trends.

**Figure S7.2.** Main model: Posterior density plot. Chain-wise posterior density plots. Similar chain densities indicate stable sampling.

**Figure S7.3.** Main model: Bulk and tail ESS. Bulk and tail effective sample size diagnostics. The dashed reference line marks ESS = 400.

**Figure S7.4.** Outcome subgroup model: Trace plot. MCMC trace plots by Markov chain. Well-mixed chains should overlap without sustained trends.

**Figure S7.5.** Outcome subgroup model: Posterior density plot. Chain-wise posterior density plots. Similar chain densities indicate stable sampling.

**Figure S7.6.** Outcome subgroup model: Bulk and tail ESS. Bulk and tail effective sample size diagnostics. The dashed reference line marks ESS = 400.

**Figure S7.7.** Population subgroup model: Trace plot. MCMC trace plots by Markov chain. Well-mixed chains should overlap without sustained trends.

**Figure S7.8.** Population subgroup model: Posterior density plot. Chain-wise posterior density plots. Similar chain densities indicate stable sampling.

**Figure S7.9.** Population subgroup model: Bulk and tail ESS. Bulk and tail effective sample size diagnostics. The dashed reference line marks ESS = 400.

**Figure S7.10.** GAM duration total: Trace plot. MCMC trace plots by Markov chain. Well-mixed chains should overlap without sustained trends.

**Figure S7.11.** GAM duration total: Posterior density plot. Chain-wise posterior density plots. Similar chain densities indicate stable sampling.

**Figure S7.12.** GAM duration total: Bulk and tail ESS. Bulk and tail effective sample size diagnostics. The dashed reference line marks ESS = 400.

| Model | Parameter | Rhat | Bulk ESS | Tail ESS |
| --- | --- | --- | --- | --- |
| Main model | b Intercept | 1.000 | 12365.43 | 13908.32 |
| Main model | sd author id:study id Intercept | 1.002 | 4137.77 | 6810.04 |
| Main model | sd author id Intercept | 1.002 | 3904.71 | 7124.10 |
| Main model | sigma | 1.000 | 16901.40 | 12624.38 |
| Outcome subgroup model | b outcome subgroupbasketball shooting accuracy | 1.000 | 15877.22 | 12940.76 |
| Outcome subgroup model | b outcome subgroupfree throw performance | 1.000 | 13900.98 | 11813.49 |
| Outcome subgroup model | b outcome subgroupfree throw time pressure | 1.000 | 19179.34 | 14716.79 |
| Outcome subgroup model | b outcome subgroupmotor coordination control | 1.000 | 12172.77 | 11165.03 |
| Outcome subgroup model | b outcome subgroupself efficacy | 1.000 | 17199.51 | 14420.05 |
| Outcome subgroup model | b outcome subgroupthree point shooting | 1.000 | 17524.60 | 13502.52 |
| Outcome subgroup model | sd author id:study id Intercept | 1.000 | 5997.10 | 8452.20 |
| Outcome subgroup model | sd author id Intercept | 1.000 | 6178.20 | 10463.31 |
| Outcome subgroup model | sigma | 1.000 | 15342.35 | 13170.87 |
| Population subgroup model | b population levelelite | 1.000 | 15597.94 | 13126.43 |
| Population subgroup model | b population levelnovice | 1.001 | 13341.07 | 11535.27 |
| Population subgroup model | b population levelrecreational | 1.000 | 14413.68 | 12187.44 |
| Population subgroup model | b population levelstudent | 1.000 | 14927.56 | 14022.63 |
| Population subgroup model | b population leveltrained | 1.000 | 14556.85 | 13231.06 |
| Population subgroup model | sd author id:study id Intercept | 1.001 | 5403.27 | 8612.96 |
| Population subgroup model | sd author id Intercept | 1.000 | 4920.58 | 7975.68 |
| Population subgroup model | sigma | 1.000 | 14911.41 | 11412.92 |
| Duration GAM model | b Intercept | 1.000 | 11997.84 | 12921.59 |
| Duration GAM model | sd author id:study id Intercept | 1.000 | 4921.92 | 7905.12 |
| Duration GAM model | sd author id Intercept | 1.000 | 5158.48 | 7832.29 |
| Duration GAM model | sds sduration weeks 1 | 1.001 | 6794.82 | 10370.24 |
| Duration GAM model | sigma | 1.000 | 18034.67 | 13411.30 |
